# Phosphorylated HPV‑16 E7 disrupts planar cell polarity in cervical cancer cells through vangl1 dysregulation

**DOI:** 10.1371/journal.ppat.1014555

**Published:** 2026-09-21

**Authors:** Ifeoluwa D. Gbala, Om Basukala, Amira Zine El Abidine, Michael P. Myers, Rebecca Bertolio, Giannino Del Sal, Lawrence Banks

**Affiliations:** 1 Tumour Virology, International Centre for Genetic Engineering and Biotechnology, Trieste, Italy; 2 Protein Networks, International Centre for Genetic Engineering and Biotechnology, Trieste, Italy; 3 Cancer Cell Signalling, International Centre for Genetic Engineering and Biotechnology, Trieste, Italy; 4 Department of Life Science, University of Trieste, Trieste, Italy; 5 IFOM ETS, the AIRC Institute of Molecular Oncology, Milan, Italy; University of Wisconsin Madison School of Medicine and Public Health, UNITED STATES OF AMERICA

## Abstract

High‑risk (HR) human papillomaviruses (HPVs) rely on the continuous expression of the E6 and E7 oncoproteins to reshape epithelial biology, drive and sustain malignant transformation. The high‑risk E7 oncoprotein is multifunctional, and its phosphorylation by Casein Kinase II (CKII) is a key post translational modification that amplifies its oncogenic potential. Therefore, it is important to extensively delineate the consequences of E7 phosphorylation on HPV-driven transformation and malignant progression. Here, we identify Vangl1, a core planar cell polarity (PCP) scaffold protein, as a novel, phosphorylation‑dependent interactor of HPV‑16 E7. Through proteomics, biochemical and cell-based assays, this study established that CKII‑mediated phosphorylation is required for Vangl1 recruitment, and that this interaction is more pronounced with HPV‑16 E7, revealing a new mechanism by which HR HPVs might disrupt epithelial organization. In CaSki cells and ectopic expression models, E7 expression profoundly disrupts Vangl1 homeostasis, creating a biphasic proteostatic imbalance in which phosphorylated Vangl1 is aberrantly stabilised. This retention parallels E7 stability, revealing a reciprocal oncogenic stabilization. Furthermore, we found that Vangl1 is depleted in cytoskeletal cell fractions in the presence of E7, and that E7 translocates to membrane compartments where it colocalizes with Vangl1. In line with this, we identified that this abnormal Vangl1 stabilization occurs from its mislocalization and impaired trafficking arising from E7’s interference with the Adaptor-Related Protein Complex 1 Subunit Mu 1 (AP1M1). Functionally, Vangl1 siRNA-mediated ablation in CaSki spheroids mirrors E6/E7 loss, leading to disrupted spheroidal architecture, reduced invasion, and increased sensitivity to chemotherapeutic stress. These phenotypes position Vangl1 as a key downstream effector through which E7 reshaped epithelial behaviour. Taken together, our findings reveal a CKII-dependent mechanism by which HPV-16 E7 hijacks a core planar polarity component to destabilize epithelial structure and promote malignant traits; thus, positioning Vangl1 as a potential druggable target in HPV-associated cervical cancer.

## Author summary

High-risk human papillomaviruses (HPVs) cause most cervical cancers. These viruses rely on the continuous expression of their E6 and E7 oncoproteins to reprogramme infected cells and promote cancer progression. Previous studies show that the oncogenic activity of E7 is strengthened by phosphorylation by Casein Kinase II (CKII), suggesting that this modification may enable E7 to engage specific host factors. In this study, we explored host cellular factors that specifically interact with the phosphorylated form of E7. We found that CKII-phosphorylated HPV-16 E7 binds to Vangl1, a key regulator of planar cell polarity. This interaction disrupts Vangl1’s normal trafficking, turnover, and localization, causing its abnormal distribution inside cells. The resulting imbalance destabilizes epithelial organization. When we reduced Vangl1 expression in HPV-positive cancer cells, they lost their invasive architecture and became more sensitive to chemotherapy similar to the effects of E6/E7 loss. Our findings reveal a novel mechanism by which HPV-16 dysregulates planar polarityto promote malignant characteristics, and identify Vangl1 modulation as a potential therapeutic approach.

## Introduction

Cervical cancer remains a major global health challenge, disproportionately affecting women in low‑ and middle‑income countries where access to screening, vaccination, and advanced treatment is limited [1,2]. Despite the availability of effective prophylactic vaccines, persistent infection with high‑risk human papillomaviruses (HPVs), particularly HPV‑16 and HPV‑18, continues to drive most cervical cancer cases worldwide [3]. These infections initiate a multistep progression from precancerous lesions to invasive carcinoma [4–6], a process sustained by the viral oncoproteins E6 and E7, which remain expressed throughout tumour development and are essential for malignant maintenance [7–11].

The HPV‑16 E7 oncoprotein is a central driver of oncogenesis. Beyond its canonical inactivation of the retinoblastoma protein (pRB) and deregulation of E2F‑dependent cell‑cycle entry [12–14], E7 undergoes post‑translational modifications that expand its oncogenic repertoire. Phosphorylation of conserved serine residues by casein kinase II (CKII) enhances E7 stability and reshapes its interactome [15,16] Yet, how phospho‑E7 engages additional host pathways to promote malignancy remains incompletely understood. This gap is particularly important because HPV‑positive tumours often display distinctive patterns of aggressive invasion and metastatic spread, suggesting that viral oncoproteins may influence cellular polarity and tissue organization [17–20].

Emerging evidence points to the non‑canonical Wnt/planar cell polarity (PCP) pathway as a critical regulator of epithelial architecture, directional migration, and metastatic behaviour [21–24]. Dysregulated PCP signalling disrupts polarity, activates Rho GTPases and JNK, and promotes epithelial‑to‑mesenchymal transition, collectively enabling invasive phenotypes across multiple cancers [24–27]. Prior studies show that HPV E6/E7 can activate PCP/JNK signalling [25,26], but the upstream viral–host interactions that initiate this activation are not known.

This unresolved link between HPV oncoproteins and PCP machinery raises a fundamental question of how HPV‑16 E7 directly engages PCP components to reprogramme epithelial behaviour and promote invasion. Here, we identify Vangl1 as a novel interactor of phosphorylated HPV‑16 E7 and demonstrate that this interaction drives Vangl1 dysregulation in cervical cancer cells. Thus, Vangl1 is a compelling candidate for this missing link, given its central role in establishing planar polarity and its oncogenic functions in migration, metastasis, and therapy resistance [28]. We show that phospho‑E7–mediated perturbation of Vangl1 enhances invasive and collective metastatic phenotypes and alters chemotherapeutic responses in three‑dimensional culture systems. These findings uncover a new mechanism by which HPV‑16 co‑opts PCP signalling to promote malignant progression and highlight Vangl1 as a potential therapeutic target in HPV‑associated cancers.

## Results

### Phosphorylated HPV‑16 E7 recruits Vangl1

**Fig 1:**
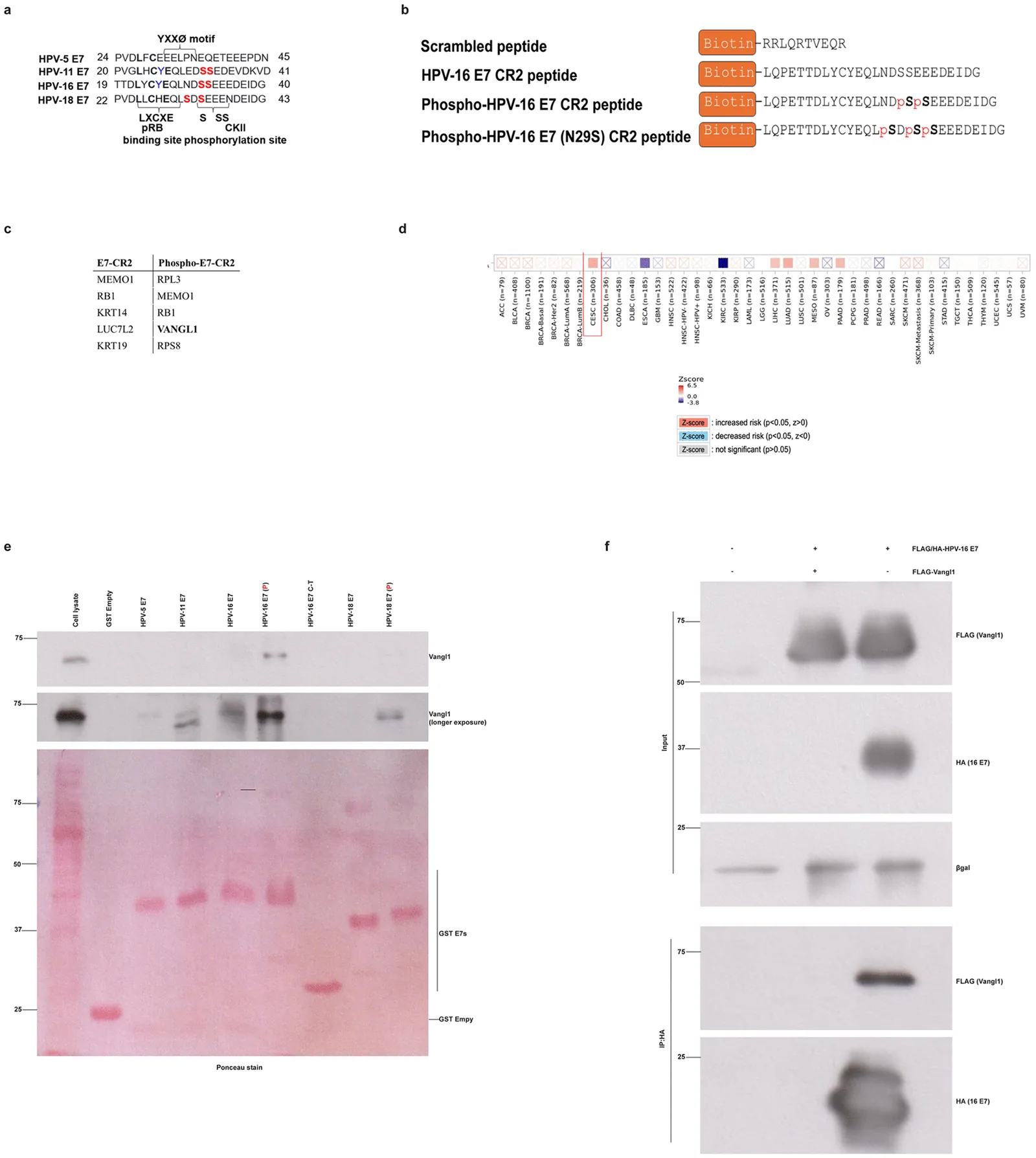
Phosphorylated HPV‑16 E7 selectively recruits Vangl1. (a) Schematic of the HPV‑16 E7 CR2 region showing the Casein Kinase II (CKII) phosphorylation site at serines 31 and 32/33. (b) Sequences of biotin‑tagged peptides corresponding to the HPV‑16 E7 CR2 region, synthesized in phosphorylated and non‑phosphorylated forms and used as bait in peptide pulldown assays. (c) Mass spectrometry analysis of HaCaT lysates pulled down with phosphorylated or non‑phosphorylated E7 peptides. The phosphorylated peptide enriched known E7 interactor pRB and selectively precipitated Vangl1. (d) TCGA analysis shows that Vangl1 expression is associated with increased clinical risk in multiple tumour types, including cervical and endocervical cancer (CESC), lung adenocarcinoma (LUAD), and pancreatic adenocarcinoma (PAAD). The statistical significance is represented as z-score and *p*-value. (e) GST pulldown assays showing strong binding of endogenous Vangl1 to phosphorylated HPV‑16 E7, with weak or undetectable binding to HPV‑11 E7, non‑phosphorylated HPV‑16 or HPV‑18 E7, phosphorylated HPV‑18 E7, or HPV‑5 E7. **‘P’** indicates phosphorylated forms of the respective E7s. (f) Co‑immunoprecipitation of FLAG‑Vangl1 with FLAG/HA‑tagged HPV‑16 E7 expressed in HEK293 cells confirms the *in vivo* interaction. Beta galactosidase (β-gal) protein expression is used as a loading control in the western blotting analyses.

Phosphorylation of high-risk HPV E7 oncoprotein by Casein Kinase II (CKII) at serine residues 31 and 32/33 is essential for maintaining the transformed phenotype in cervical cancer–derived cell lines [15,16,29]. Given the importance of this modification, we sought to identify phospho-specific binding partners of HPV-16 E7 and to determine the functional relevance of such interactions. To this end, we synthesized biotin-tagged peptides corresponding to the CKII site within the CR2 region of HPV-16 E7 (Fig 1a), in both phosphorylated and non-phosphorylated forms (Fig 1b). The peptides were then used as bait in streptavidin pulldown assays with HaCaT keratinocyte lysates; the bound proteins were analysed by mass spectrometry.

Proteomic analysis showed that the canonical E7 interactor, pRB was one of the most enriched proteins, confirming the relevance of the assay. Consistent with previous reports [30], the phosphorylated E7 peptide recovered more pRB derived peptides than the non-phosphorylated peptide (Fig 1c), supporting the established role of negative charge at the CKII site in enhancing E7–pRB binding. Of the other proteins precipitated, Vangl1 was notable, for being selectively enriched by the phosphorylated HPV-16 E7 peptide (Fig 1c).

Vangl1 is a core planar cell polarity (PCP) protein that functions as a scaffold in the Wnt/PCP pathway and is essential for cell orientation and migration [28]. To explore the potential clinical relevance of this interaction, we examined Vangl1 expression in different cancers as published in The Cancer Genome Atlas (TCGA). Elevated Vangl1 gene expression was significantly associated with increased risk in six tumour types, including cervical and endocervical cancer (CESC), lung adenocarcinoma (LUAD), and pancreatic adenocarcinoma (PAAD) (Fig 1d).

We next investigated whether Vangl1 physically interacts with E7 proteins from high-risk (HPV-16, HPV-18), low risk (HPV-11) and cutaneous (HPV-5) HPV types. GST pulldown assays using HaCaT lysates revealed strong binding of endogenous Vangl1 to phosphorylated HPV-16 E7, whereas only weak or undetectable binding was observed with HPV-11 E7, non-phosphorylated HPV-16 or HPV-18 E7, phosphorylated HPV-18 E7, or HPV-5 E7 (Fig 1e). Vangl1 did not bind the HPV-16 E7 C-terminus (residues 41–98), indicating that the N-terminal half mediates this interaction.

To validate the interaction *in vivo*, FLAG-tagged Vangl1 and FLAG/HA-tagged HPV-16 E7 were co-expressed in HEK293 cells. Immunoprecipitation with anti-HA beads confirmed that Vangl1 associates with HPV-16 E7 in cells (Fig 1f, S1 Fig). Together, these findings identify Vangl1 as a novel, phosphorylation-dependent interactor of HPV-16 E7.

### CKII phosphorylation allows selective E7–Vangl1 binding

**Fig 2:**
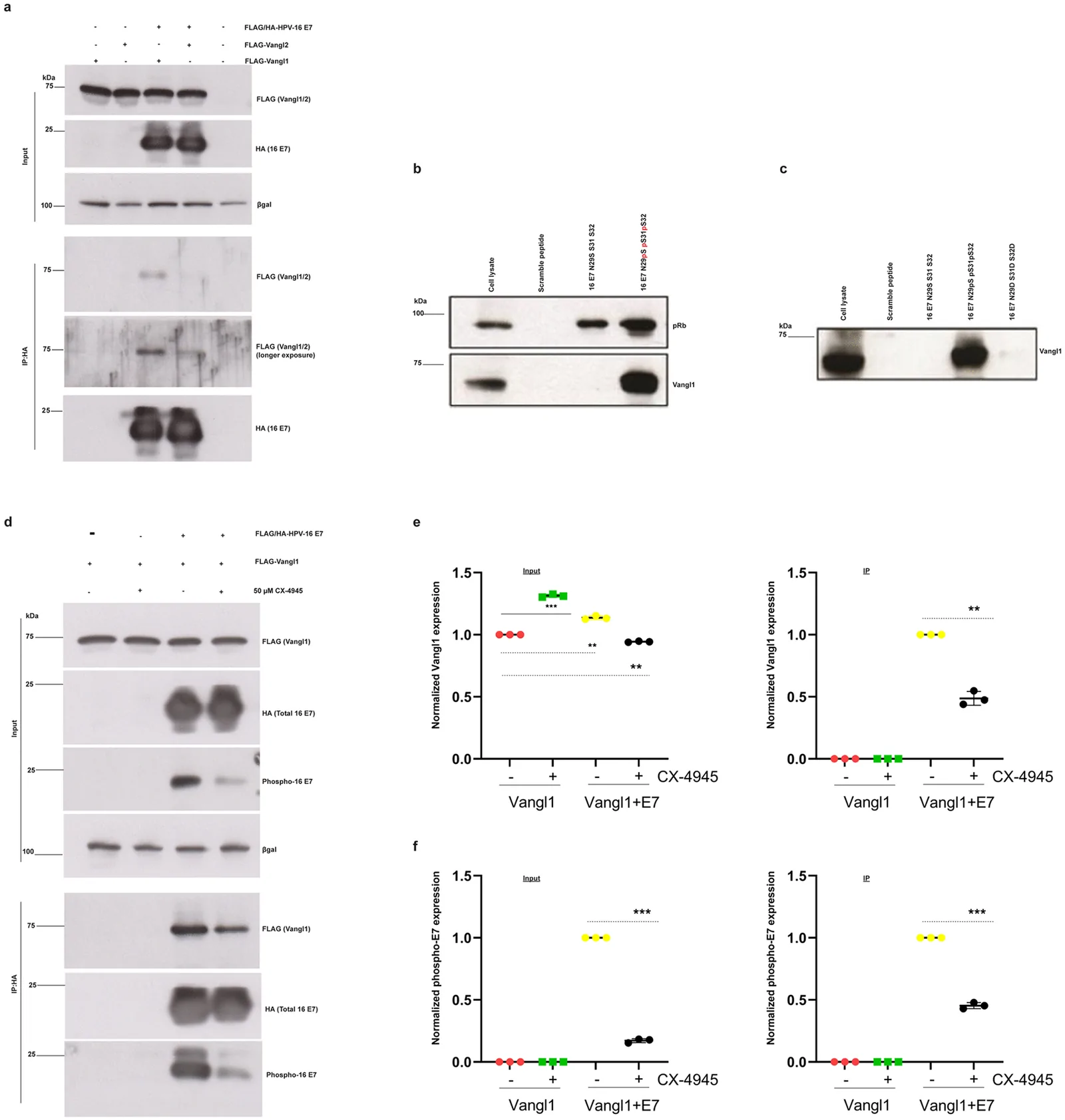
CKII phosphorylation enables HPV‑16 E7 to bind specifically to Vangl1. (a) Co‑immunoprecipitation of FLAG‑tagged Vangl1 or Vangl2 with FLAG/HA‑tagged HPV‑16 E7 expressed in HEK293 cells. HPV‑16 E7 binds strongly to Vangl1 but only minimally to Vangl2. (b) Peptide pulldown assays using phosphorylated and non‑phosphorylated HPV‑16 E7 N29S CR2 peptides. Vangl1 binds strongly to the phosphorylated N29S peptide but not to its non‑phosphorylated form. (c) Pulldown assays using phosphomimic HPV‑16 E7 CR2 peptides in which CKII‑targeted serines were substituted with aspartic acid. The phosphomimic fails to bind Vangl1. ‘**p’** indicates phosphorylation. (d) Co‑immunoprecipitation of FLAG‑Vangl1 with FLAG/HA‑tagged HPV‑16 E7 in HEK293 cells treated with the CKII inhibitor CX‑4945. CKII inhibition markedly reduces Vangl1–E7 association. Beta galactosidase (β-gal) protein expression is used as a loading control in the western blotting analyses. (e) Vangl1 band densities of input and co-immunoprecipitation samples were normalized to the β-gal band density, and the data were used to plot densitometry graphs showing that CX-4945 treatment significantly reduces the amount of Vangl1 bound by HPV-16 E7. (f) Phospho-E7 band densities of input and co-immunoprecipitation samples were normalized to the β-gal band density, and the data were used to plot densitometry graphs showing that CX-4945 treatment significantly reduces phosphorylated E7 levels. Data is shown as the fold changes of normalized Vangl1 levels relative to the control (Vangl1 only or untreated Vangl1 + E7) ± standard deviation. *p* values of 3 independent experiments were calculated by Student’s t test. (*p* values of 3 independent experiments were calculated by Student’s t test. (*: *p*-value < 0.05; **: *p*-value <0.01; ***: *p*-value <0.001).

Because Vangl1 and its paralog Vangl2 share 72% identity and overlapping roles in Wnt/PCP signalling [28], we examined whether HPV-16 E7 might also interact with Vangl2. Co-immunoprecipitation assays from HEK293 cells expressing FLAG-tagged Vangl1 or Vangl2 with FLAG/HA-tagged HPV-16 E7 showed that E7 binds robustly to Vangl1, but only minimally to Vangl2 (Fig 2a), demonstrating paralog-specific selectivity.

To further assess the role of CKII phosphorylation, we performed peptide pulldown assays using the HPV-16 E7 N29S variant, which undergoes enhanced CKII phosphorylation [29]. Vangl1 bound strongly to the phosphorylated N29S peptide but not to its non-phosphorylated counterpart (Fig 2b). Notably, the substitution of the CKII-targeted serines with aspartic acid to generate phospho-mimic peptides failed to recapitulate binding (Fig 2c), indicating that negative charge alone is insufficient and that true phosphorylation is required.

We next tested whether CKII activity is required for the interaction *in vivo*. To do this, HEK293 cells co-expressing Vangl1 and HPV-16 E7 were treated with the pharmacological CKII inhibitor CX-4945. CKII inhibition markedly reduced Vangl1 co-immunoprecipitation with E7 (Fig 2d-e), confirming that CKII-mediated phosphorylation of E7 is essential for Vangl1 binding. As expected, CKII inhibition reduced phosphorylated 16-E7 levels in the cells (Fig 2d and f). Taken together, these results demonstrate that HPV-16 E7 selectively binds Vangl1 in a CKII phosphorylation-dependent manner.

### HPV E7 expression modulates Vangl1 protein abundance in cervical cancer cells

Having established that Vangl1 is a phosphorylation-specific substrate of E7, we wanted to determine how Vangl1 behaves in HPV‑positive cervical cancer cells. For this, we examined Vangl1 protein levels following siRNA‑mediated knockdown of E6/E7 or Vangl1 in HPV‑positive CaSki and HeLa cells, HPV‑negative C33-A cells, and non‑transformed HaCaT keratinocytes. It is important to mention that Vangl1 appears as two bands on the blots in most cases – the lower band is unphosphorylated Vangl1 and the upper band is the phosphorylated form.

**Fig 3:**
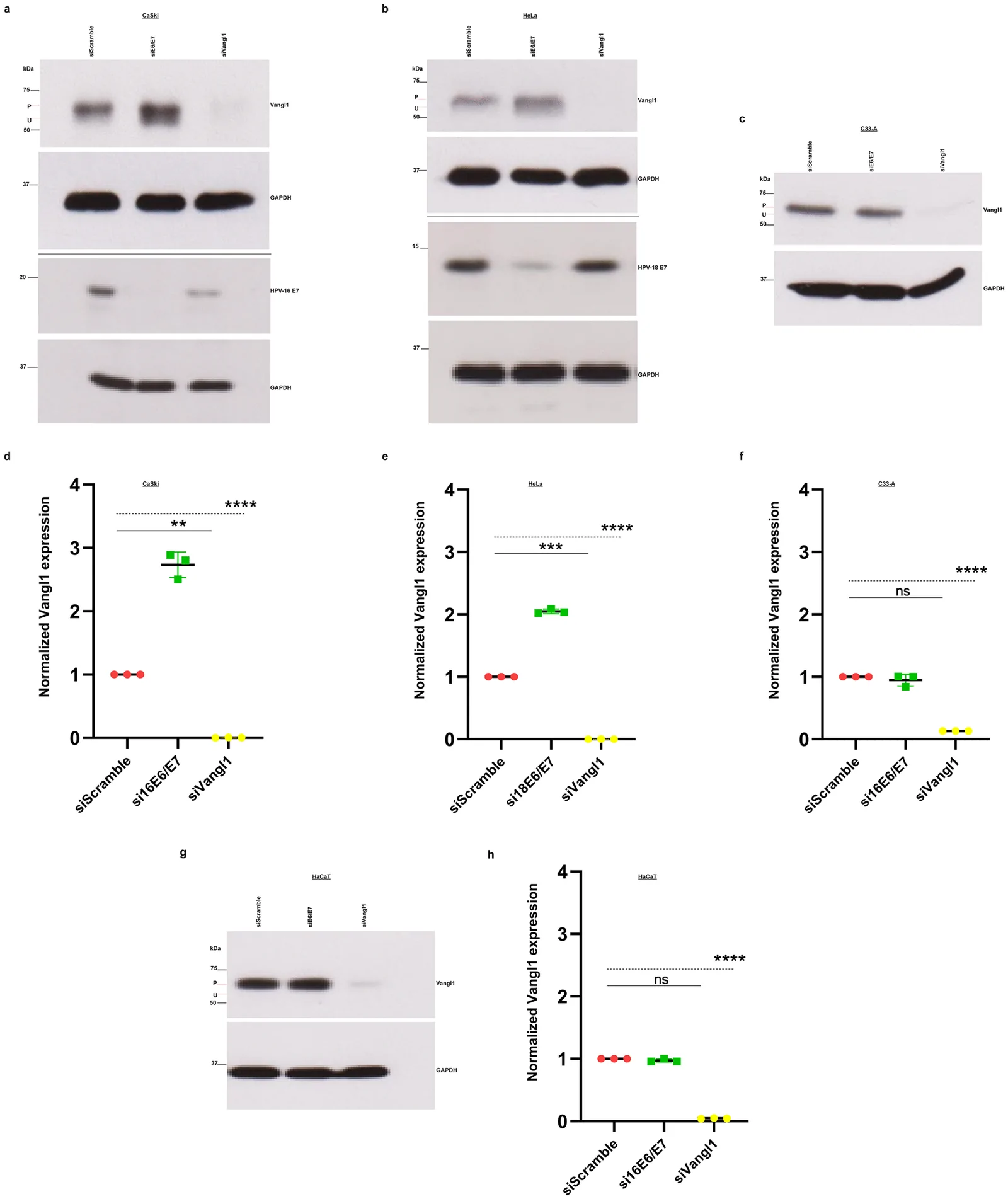
HPV E7 expression modulates Vangl1 protein abundance. Immunoblot analysis of Vangl1 and E7 protein levels following siRNA‑mediated knockdown of E6/E7 or Vangl1. (a, b) In HPV‑16 E7-positive CaSki cells and HPV‑18 E7-positive HeLa cells, E6/E7 depletion increases total Vangl1 abundance. (c, d) In HPV‑negative C33-A cells and non‑transformed HaCaT keratinocytes, Vangl1 remains unchanged following E6/E7 knockdown. GAPDH expression is shown as loading control. Vangl1 band density was normalized to GAPDH band density, and the data was used to plot the densitometry graphs for all cell lines (d, e, g, h). Data is shown as the fold-changes of normalized Vangl1 levels relative to the control (siScramble) ± standard deviation. *p* values of 3 independent experiments were calculated by Student’s t test. (*: *p*-value < 0.05; **: *p*-value <0.01; ***: *p*-value <0.001).

It can be seen in Fig 3a and b, that the protein levels of unphosphorylated Vangl1 (lower band) in siScramble-treated CaSki and HeLa cells are very low but increase upon E6/E7 knockdown in both cell lines increasing total Vangl1 levels relative to the control (Fig 3d and e). It is also notable that, Vangl1 depletion modestly reduced HPV-16 E7 abundance in CaSki cells but had no effect on HPV-18 E7 in HeLa cells, which might reflect the weaker physical interaction between HPV‑18 E7 and Vangl1. In contrast, the majority of Vangl1 was phosphorylated in HPV‑negative C33-A and HaCaT cells, and its level remained unchanged upon siE6/E7 treatment (Fig 3c, f-h), confirming that the increase in Vangl1 levels upon E6/E7 knockdown is specific to HPV‑positive cervical cancer cells and not an off‑target effect of siRNA treatment. To confirm that the upper bands were indeed phosphorylated forms of Vangl1, we performed a lambda phosphatase treatment on whole cell lysates of CaSki, HeLa, C33-A and HaCaT cells. As shown in S2 Fig, lambda treatment caused a shift in mobility of the upper Vangl1 band, thus increasing the pool of the unphosphorylated (lower) Vangl1 pool. This confirms that the upper band is indeed phosphorylated Vangl1.

These findings suggest that E7 expression perturbs Vangl1 protein homeostasis in HPV-transformed cervical cancer cells and that Vangl1 may contribute to the stability of HPV‑16 E7.

### E7 selectively prolongs half‑life of phosphorylated Vangl1

**Fig 4:**
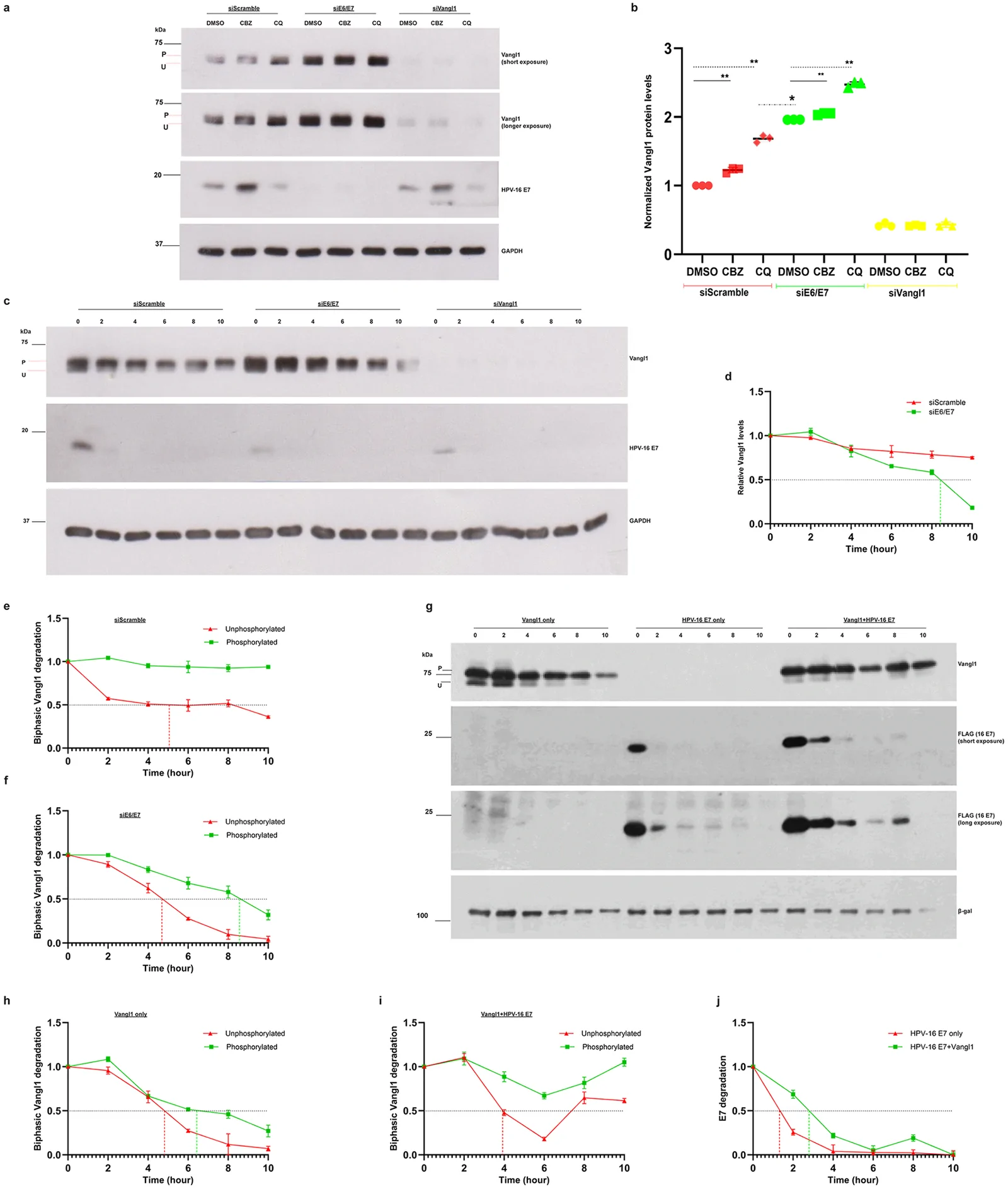
E7 prolongs Vangl1 half‑life through a non‑canonical stabilization mechanism. **(a)** Immunoblot analysis of Vangl1 in CaSki cells transfected with control, Vangl1 or E6/E7 siRNAs and treated with the proteasome inhibitor CBZ, the lysosomal inhibitor chloroquine (CQ), or DMSO. E6/E7 knockdown alone increases Vangl1 levels more strongly than either inhibitor. Data is shown as the fold changes of normalized Vangl1 levels relative to the control (siScramble DMSO) ± standard deviation. *p* values of 3 independent experiments were calculated by Student’s t test. *: *p*-value < 0.05; **: *p*-value <0.01; ***: *p*-value <0.001. **(b)** Western blot of cycloheximide chase assays in CaSki cells shows that total Vangl1 half‑life in control cells is longer than in E7‑depleted cells. GAPDH expression is shown as loading control. Vangl1 band density was normalized to GAPDH band density, and the data was used to plot densitometry graphs for the half-life of total Vangl1 **(c)**, and the bi-phasic degradation of phosphorylated and unphosphorylated Vangl1 in siScramble (d) and siE6/E7 (e) cells. Data is shown as the fold changes of normalized Vangl1 levels relative to the control (siScramble) ± standard deviation. The green dashed line represents the half-life of total Vangl1 while the red dashed line represents the half-life of unphosphorylated Vangl1 **(f)** Western blot of cycloheximide chase assays in HEK293 cells overexpressing Vangl1 with or without HPV‑16 E7 shows a reciprocal stabilization of E7 and Vangl1 when they are co-expressed. β -gal expression is shown as loading control. Vangl1 band density was normalized to the β-gal band density, and the data was used to plot densitometry graphs of the bi-phasic degradation of phosphorylated and unphosphorylated Vangl1 in cells expressing Vangl1 only (g) or in co-expression with HPV-16 E7 **(j)**, as well as half-life of total E7. Dotted lines on time-kinetic graphs indicate specific half-life. Data is shown as the fold changes of normalized Vangl1 levels of 3 independent experiments relative to the control (Vangl1 only) ± standard deviation.

The accumulation of Vangl1 seen in the HPV-positive cervical cancer cell lines prompted us to ask whether E7 might enhance Vangl1’s proteasomal or lysosomal degradation, pathways previously described for its natural turnover [31]. CaSki cells transfected with either siScramble or siE6/E7 were treated with the proteasome inhibitor CBZ, the lysosome inhibitor chloroquine (CQ), or DMSO. We found that, in control-treated cells, both inhibitors modestly increased Vangl1 levels, with CQ having a stronger effect (Fig 4a). Notably, there was a selective increase in the lower (unphosphorylated) Vangl1 band upon CQ treatment, suggesting that unphosphorylated Vangl1 may be more susceptible to lysosomal degradation, and this is consistent with previous reports [22]. However, E6/E7 knockdown in CaSki cells increased Vangl1 levels more strongly than inhibitor treatment in siScramble-treated cells. Furthermore, combining E6/E7 knockdown with CQ treatment did not further increase Vangl1 levels, which argues against a model tightly controlled by E7-enhanced lysosomal degradation. These findings suggest that E7 may contribute to the lysosomal degradation of Vangl1, but do not solely explain the accumulation of Vangl1 upon E7 knockdown, suggesting that other mechanisms may also be in play.

To investigate this, we performed Cycloheximide chase assays to monitor the posttranslational stability of Vangl1 in the presence of E7. In control-treated CaSki cells, the half‑life of total Vangl1 exceeded 10-h but decreased to 8.4-h upon E6/E7 knockdown (Fig 4b). This was intriguing because the pool of total Vangl1 in E6/E7-depleted CaSki cells is at least twice that of control-treated cells across different experiments. Strikingly, the control-treated cells exhibited a biphasic degradation pattern (Fig 4c), unlike the siE6/E7-treated cells where steady concurrent degradation was seen (Fig 4d). In Fig 4c, we show that unphosphorylated Vangl1 (lower band labelled ‘U’) was initially degraded rapidly, reaching its half-life in the first 5-h and then remained stable until the 8^th^ hour followed by a decline. This aligns with our earlier observation that CQ treatment selectively rescued unphosphorylated Vangl1 in siScramble-treated cells. In contrast, the half-life of the phosphorylated form (upper band labelled ‘P’) exceeded 10-h, much longer than the reported 3–6-h in normal cells [31], indicating that phosphorylated Vangl1 undergoes aberrant proteostasis in HPV-transformed cervical cancer cells. Together, these observations indicate that the increase in total Vangl1 pool following E7 loss is not the result of enhanced stability *per se*, but instead reflects a non‑canonical regulatory mechanism.

To validate this, we overexpressed Vangl1 with or without HPV‑16 E7 in HEK293 cells. Total Vangl1 half‑life increased from 4 - 5 h to over 10 h when co‑expressed with E7 (S3 Fig). As in the CaSki siScramble group, HEK293 cells co-expressing Vangl1 and HPV-16 E7 exhibited bi-phasic degradation, whereby the half-life of unphosphorylated Vangl1 was reached in in about 4 h while the half-life of phosphorylated Vangl1 exceeded 10 h (Fig 4f-h). After 4 h, the unphosphorylated Vangl1 pool showed an irregular pattern of abundance, a trend also seen in phosphorylated Vangl1 across the timepoints (Fig 4f-h). In contrast, cells expressing Vangl1 only, without E7 expression, showed a concurrent steady decline in both Vangl1 pools, similar to the siE6/E7 CaSki cells. Unphosphorylated Vangl1 reached its half-life around 5 h and the phosphorylated pool at around 6.8 h (Fig 4f-h). Remarkably, E7’s half‑life increased by two-fold when co‑expressed with Vangl1 (Fig 4f and i). In addition to the difference in half-life, E7 remained detectable 8 h post cycloheximide treatment when co-expressed with Vangl1 while it was no longer visible after the 2^nd^ hour of treatment when expressed alone (Fig 4f and i). Taken together, these results reveal a reciprocal stabilization mechanism in which E7 aberrantly prolongs the half‑life of phosphorylated Vangl1 for its stabilization.

### E7 alters Vangl1 localization in cervical cancer cells

**Fig 5:**
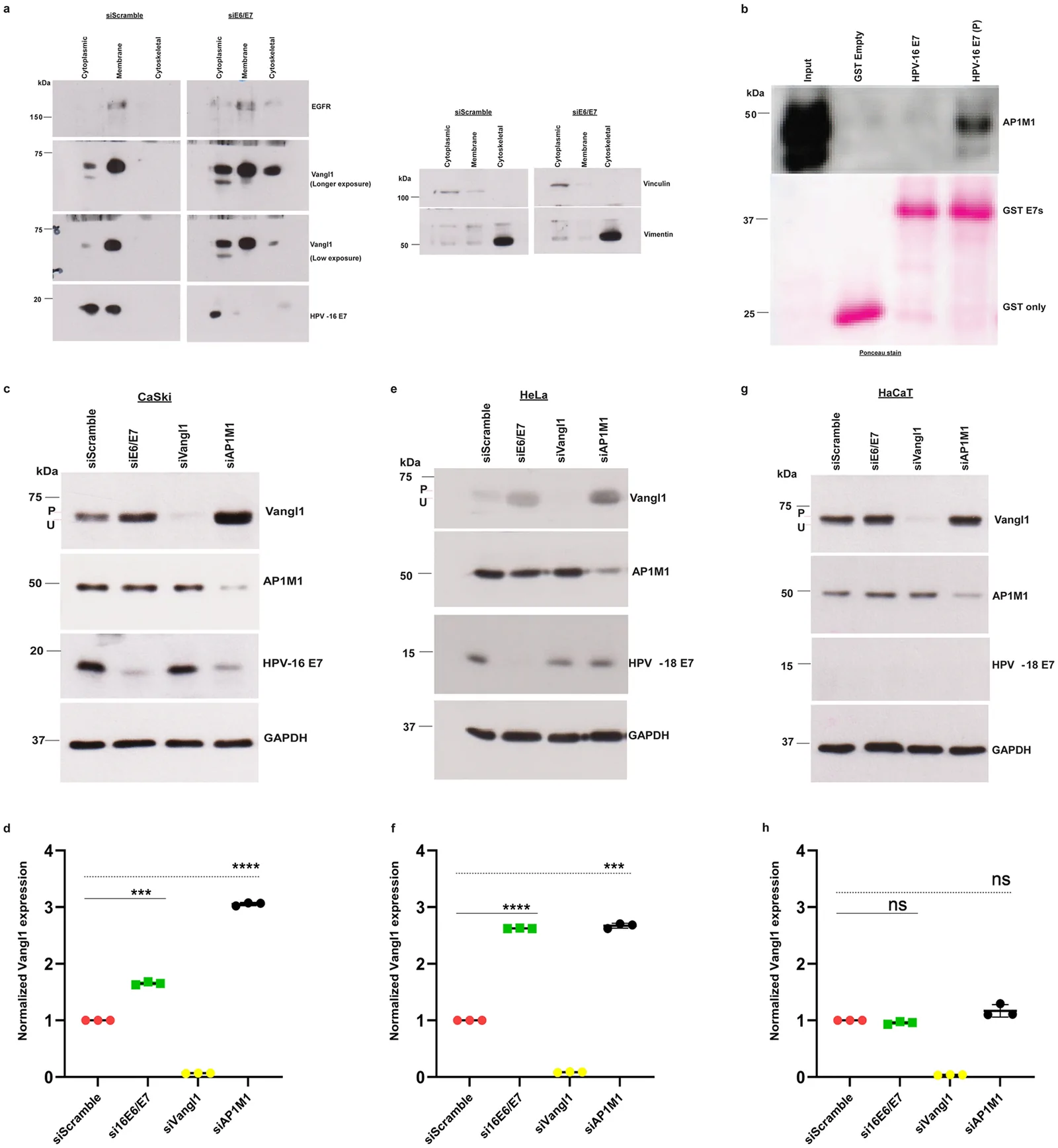
E7 remodels AP1M1‑dependent trafficking to alter Vangl1 localization. (a) Subcellular fractionation of CaSki cells transfected with control or E6/E7 siRNAs. Vangl1 is absent from the cytoskeletal fraction in control cells but becomes detectable upon E6/E7 knockdown. (b) GST pulldown assays showing that AP1M1 selectively binds phosphorylated HPV‑16 E7. (c,e) Immunoblot analysis of CaSki and HeLa cells shows that Vangl1 abundance increases upon E6/E7 depletion and is further increased by AP1M1 knockdown. (g) Immunoblot analysis of HaCaT keratinocytes shows that neither E6/E7 knockdown nor AP1M1 knockdown results in increased Vangl1 levels. GAPDH expression is shown as loading control. Vangl1 band density was normalized to GAPDH band density, and the data was used to plot densitometry graphs for the cell lines (d,f,h). Data is shown as the fold changes of normalized Vangl1 levels relative to the control (siScramble) ± standard deviation. *p* values of 3 independent experiments were calculated by Student’s t test. *: *p*-value < 0.05; **: *p*-value <0.01; ***: *p*-value <0.001.

Because protein sequestration, aggregation and mislocalization are established mechanisms by which protein turnover and overall protein abundance are altered [32,33], we examined whether E7 might affect Vangl1 subcellular distribution within cervical cancer cells. Subcellular fractionation of CaSki cells showed that no Vangl1 was present in the cytoskeletal fraction in control cells, but that phosphorylated Vangl1 relocates there upon E6/E7 knockdown (Fig 5a). In addition, there is an increase in the levels of both unphosphorylated Vangl1 and phosphorylated Vangl1 pools in the cytoplasmic fraction upon E6/E7 knockdown, whereas membrane‑associated Vangl1 remained largely unchanged. These findings suggest that Vangl1 trafficking and metabolism is impaired in the presence of E6/E7, reflected by limited export or enhanced cytoplasmic instability, prolonged retention at the membrane, and no localization to the cytoskeletal regions.

Vangl1 trafficking is regulated by the μ1 subunit of the AP-1 clathrin adaptor complex (AP1M1), which mediates sorting between the trans-Golgi network, plasma membrane, cytoskeleton and lysosomes [34–36]. Given AP1M1’s central role in Vangl1 trafficking, we asked whether AP1M1 might participate in the E7-driven regulation of Vangl1. First, we used GST pulldown assays to determine whether Vangl1 interacts physically with AP1M1. These showed that phosphorylated, but not unphosphorylated HPV-16 E7 binds to AP1M1 (Fig 5b), mirroring the Vangl1 interaction pattern.

We then wanted to assess the dynamics of Vangl1, AP1M1, and E7 interactions in cervical cancer cell lines and in normal keratinocytes. We had found earlier (Fig 3), that E6/E7 knockdown increases Vangl1 abundance in whole cell lysates of CaSki and HeLa cells; and it can be seen in Fig 5c-f that, this effect was even more pronounced when AP1M1 is also depleted. Notably, AP1M1 knockdown also led to a substantial reduction in E7 levels in both cell lines, indicating that HPV‑18 E7 uses a similar mechanism as HPV‑16 E7, to modulate Vangl1, despite its weaker physical interaction. Collectively, these findings suggest; first, that E7 probably sequesters AP1M1, altering the sorting and localization of Vangl1, such that loss of E7 restores proper trafficking; and second, that depletion of AP1M1 destabilizes E7, effectively shutting down the impaired trafficking and its associated cytoplasmic instability, which in turn reduces Vangl1 turnover. Both suggestions point to AP1M1 as a contributing factor, enabling E7 to manipulate Vangl1 homeostasis. In contrast, neither E6/E7 knockdown (as control) nor AP1M1 knockdown led to an increase in Vangl1 in HaCaT keratinocytes (Fig 5g and h), indicating that this regulatory axis is specific to HPV-transformed cervical cancer cells.

Further delineating this relationship, subcellular fractionation of CaSki cells following AP1M1 knockdown revealed Vangl1 accumulation across cytoplasmic, membrane, and cytoskeletal fractions (Fig 5f), closely resembling the pattern observed with E6/E7 knockdown. Collectively, these findings indicate that E7 expression negatively regulates Vangl1 sorting and trafficking in HPV-transformed cervical cancer cells.

### Vangl1 depletion disrupts 3D spheroid integrity

**Fig 6:**
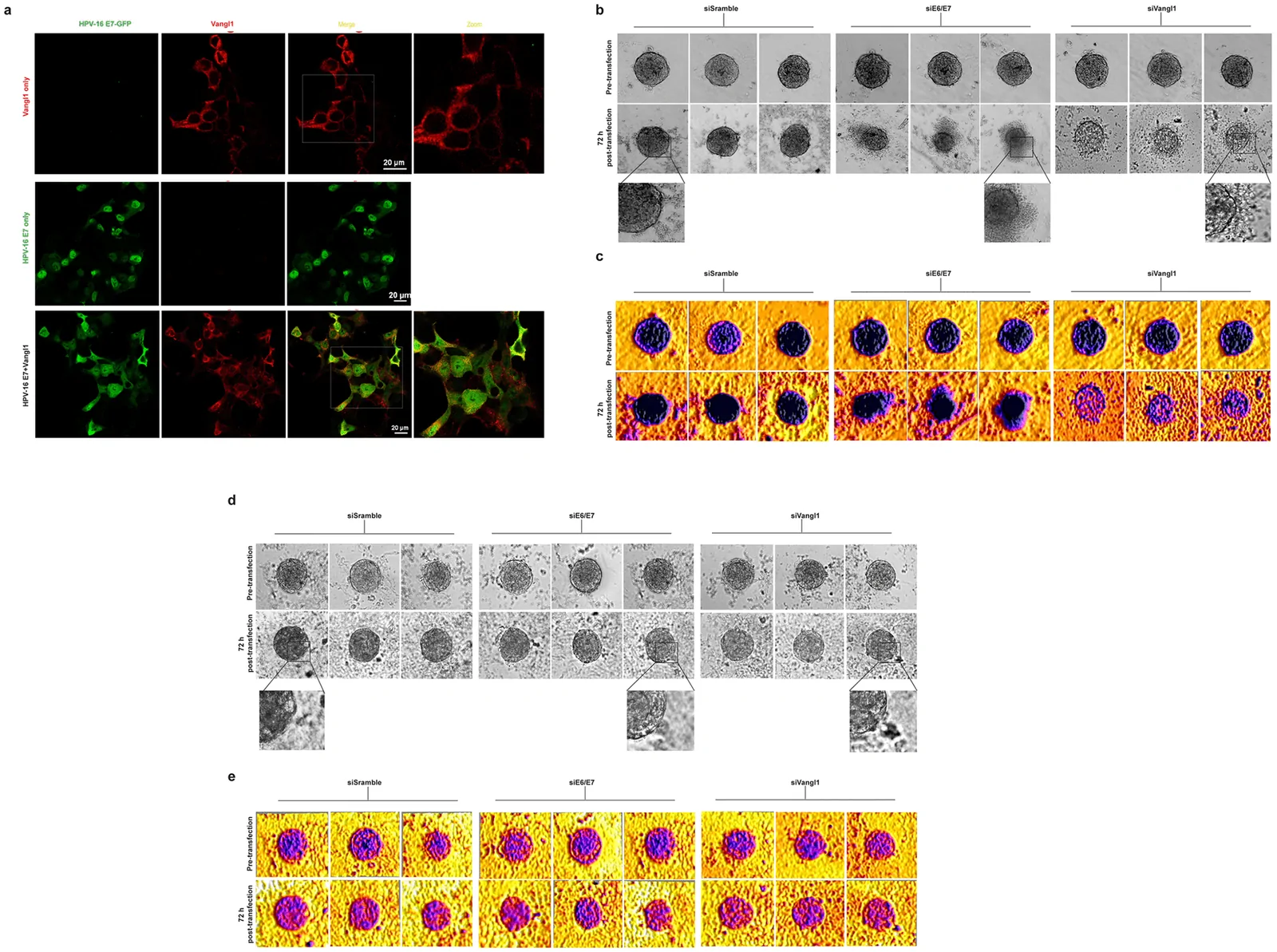
Vangl1 depletion disrupts 3D spheroid integrity. (a) Confocal microscopy of HEK293 cells expressing Vangl1 alone, HPV‑16 E7 alone, or both proteins together, show diffuse, cytoplasmic Vangl1 with reduced cortical levels in cells expressing E7. Merged and zoomed images show co‑localization of Vangl1 and E7 within intracellular compartments. (b) Brightfield images of CaSki spheroids following siRNA‑mediated knockdown of E6/E7 or Vangl1 showing spheroids with pronounced structural disintegration, irregular edges, and reduced cohesion. (c) 3D surface plots of CaSki spheroids highlight architectural defects. Control spheroids show uniform signal intensity and well‑defined boundaries, while E6/E7 and Vangl1 knockdown spheroids display diffuse signal, fragmented contours, and disrupted core organization. (d) Brightfield images of HaCaT spheroids before and after siRNA‑mediated knockdown of E6/E7 or Vangl1 showing relatively intact spheroids without evident structural disintegration. (e) 3D surface plots of HaCaT spheroids highlight intact spheroidal architecture. The colour‑intensity scale bars accompanying the 3D plots function as legends, where increasing colour intensity corresponds to higher cellular presence or signal density of the spheroids and within the spheroid environment. Higher peaks and warmer colours indicate regions of cell accumulation, while lower plateaus and cooler colours reflect reduced cellular density. Micrographs were prepared using the Quickfigures plugin of ImageJ and the brightness of the panels was uniformly enhanced post image processing.

To visualize the spatial dynamics of Vangl1 in the presence of HPV‑16 E7 in light of our findings in Fig 5, we performed immunofluorescence staining in HEK293 cells expressing Vangl1 alone, HPV‑16 E7 alone, or both proteins together. As shown in Fig 6a, cells expressing Vangl1 alone exhibit a structured, peripheral distribution of Vangl1 (red fluorescence of Vangl1) consistent with membrane and cytoskeletal localization. In contrast, co-expression of Vangl1 and HPV‑16 E7 (green fluorescence) markedly altered Vangl1 localization. Vangl1 becomes more diffuse and cytoplasmic, with reduced levels in the cortex. E7 expressed alone appears to be predominantly nuclear, but with visible cytoplasmic pools that become more concentrated upon co-expression with Vangl1. Strikingly, some cells (red arrows) show a dramatic redistribution of E7 from the nucleus to the peripheral compartments, where it co-localizes (regions of yellow fluorescence) with Vangl1. The merged and zoomed panels highlight the co-localization of Vangl1 and E7 in intracellular compartments. These observations indicate that HPV-16 E7 is recruited to subcellular compartments where Vangl1 is present, resulting in aberrant distribution of Vangl1. These data, together with the biochemical assays, suggest a model in which Vangl1 is sequestered in cervical cancer cells, impairing its scaffold function and contributing to its defective turnover.

We then wanted to know how this mislocalization might impact Vangl1 function in a physiologically relevant context [37]. To do this, we examined Vangl1’s role in cervical cancer spheroids, where the 3D architecture more faithfully preserves the polarity cues. For polarity proteins, mislocalization can have significant implications, including disruption of normal cell architecture, signalling, and tissue organization, potentially contributing to pathological states [38–40]. To assess these functional consequences, we examined spheroid morphology in HPV-positive (CaSki) cells and normal keratinocytes (HaCaT) cells following siRNA‑mediated knockdown of E6/E7 or Vangl1. Brightfield imaging revealed that control CaSki spheroids (siScramble) maintained their compact, rounded architecture over 72 h (Fig 6b). In contrast, CaSki spheroids subjected to E6/E7 exhibited marked structural disintegration, extensive cell dissociation, and reduction of spheroid size, all indicative of loss of cell viability. This phenotype reiterates that the loss of E6 and E7 oncoproteins induces apoptotic death in HPV-positive cervical cancer cells [41–44]. Similar to the siE6/E7 phenotype, Vangl1 depletion results in structural disintegration. The spheroids showed irregular edges due to cell blebbing and continuous loss of cells on the spheroidal edge.

To further contextualize the structural differences across groups, 3D surface plots were generated in ImageJ to enhance visualization of spheroid architecture. These reconstructions create a topographical landscape, allowing volumetric features such as core compaction, peripheral spreading, and structural irregularities to be enhanced beyond what is visible in 2D brightfield projections. As shown in Fig 6c, the plots show uniform signal intensity and well‑defined boundaries of control spheroids, indicating the maintenance of compactness and cell viability. The siE6/E7 plots show distorted spheroids and a decreasing colour gradient from the spheroidal core (dark purple) to fragmented layers of cells (lighter purple shades), indicating decrease in cellular density and loss of compactness. Whereas, the Vangl1 knockdown spheroids showed diffuse and varying signal intensities, fragmented contours on spheroid edges, and disrupted core organization, indicating disruption of structural integrity (Fig 6c). In contrast to the observed phenotypes of CaSki spheroids upon E6/E7 or Vangl1 knockdown, HaCaT spheroids showed no cell dissociation, edge disintegration or any notable morphological changes compared with the siScramble control (Fig 6d and e). This indicates that the phenotypes of CaSki spheroids upon E6/E7or Vangl1 ablation are specific to the cells’ oncogenic state.

Together, these data show that the continuous expression of E6/E7 is crucial for the viability and structural maintenance of the spheroids. The parallel phenotype of the Vangl1-depleted CaSki spheroids, albeit less pronounced, suggests that Vangl1 is a critical downstream effector of E7 in maintaining essential epithelial architecture and integrity. Thus, the spheroid models emphasise that E7‑driven Vangl1 mislocalization is essential for preserving a favourable oncogenic structure in transformed cervical cancer cells.

### Vangl1 supports collective invasion and matrix‑responsive architecture in transformed cervical cancer cells

**Fig 7:**
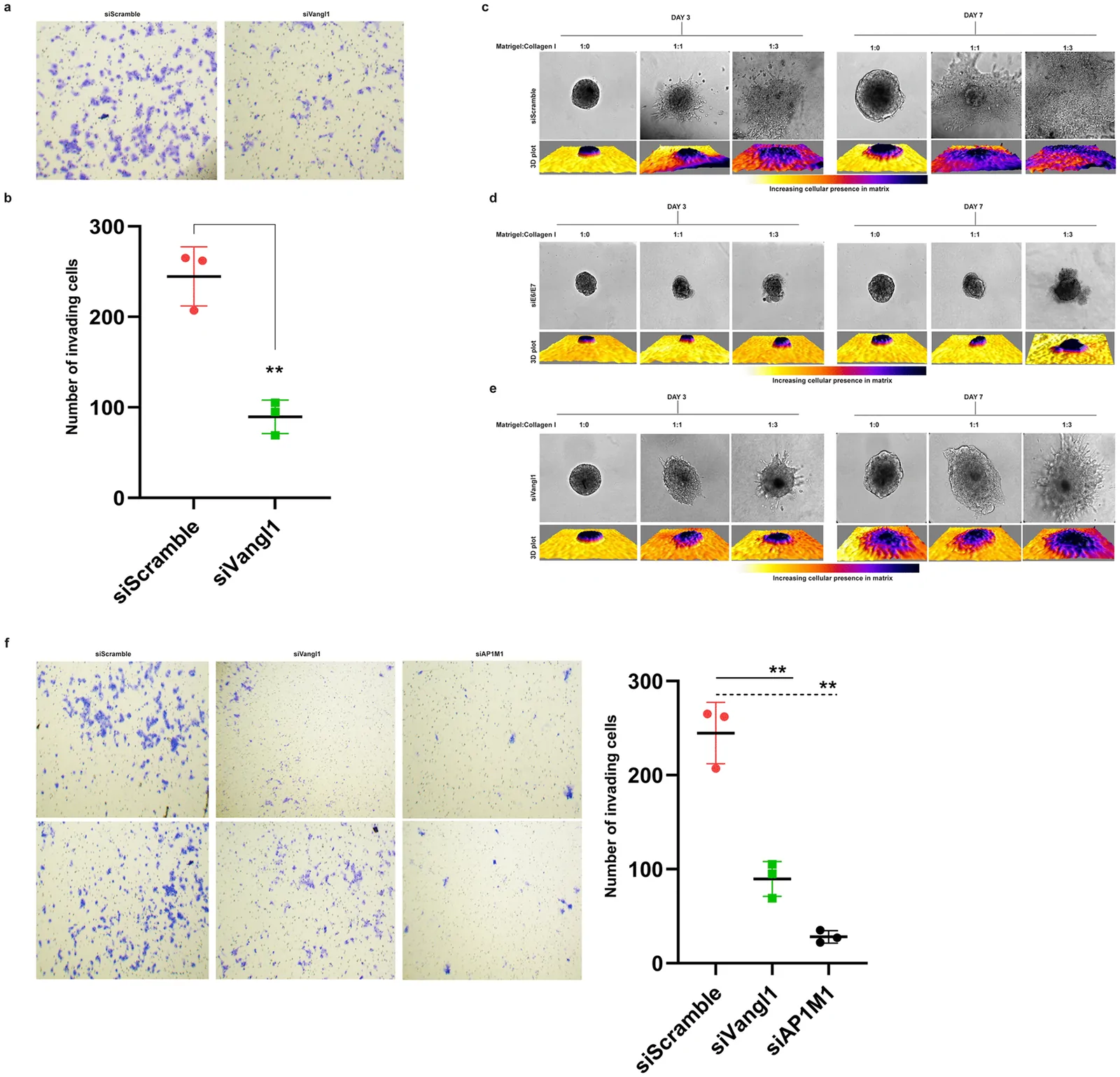
Vangl1 is essential for maintaining invasive phenotype in cervical cancer cells. (a) Brightfield representative images from 2D Matrigel invasion assays in CaSki cells show dense invasion in control cells, whereas Vangl1‑depleted cells show markedly reduced invasion. (b) Quantification of invading cells using the ImageJ Cell counter tool confirms a significant decrease in invasion upon Vangl1 knockdown. (c) Brightfield images of spheroid invasion across increasing collagen content (Matrigel:Collagen 1:1 and 1:3) show collective invasion in control spheroids; (d) E6/E7‑depleted spheroids fail to generate organized invasive protrusions in either matrix condition; (e) Vangl1‑depleted spheroids similarly lack robust invasive structures and display matrix‑dependent architectural defects. Corresponding 3D surface plots and colour‑intensity scale bars reflect invasiveness, where increasing colour intensity corresponds to higher cellular presence or signal density within the spheroid environment. Higher peaks and warmer colours indicate regions of cell accumulation, while lower plateaux and cooler colours reflect reduced cellular density. Invasiveness of the spheroids was monitored for 7 days and imaged at 20x magnification. (f) Brightfield representative images from 2D Matrigel invasion assays following AP1M1 knockdown reveal a pronounced reduction in invasion. Invading cells in the 2D assays were stained with crystal violet and imaged using a brightfield microscope at 10x magnification. *p* values of 3 independent experiments were calculated by Student’s t test. *: *p*-value < 0.05; **: *p*-value <0.01; ***: *p*-value <0.001; ****: *p*-value <0.0001. Micrographs were prepared using the Quickfigures plugin of ImageJ. The brightness of the panels was uniformly enhanced post-image processing.

Having found that Vangl1 is important for maintaining epithelial architecture in CaSki spheroids, we next investigated the implication of this structural role. The Wnt/PCP pathway is well established as a regulator of cell migration and apical-basal coordination [45], and Vangl dysregulation has been linked to metastatic progression [24,46,47]. We therefore asked whether Vangl1 could contribute to invasive behaviour in transformed cervical cancer cells. In a 2D Matrigel invasion assay, siRNA‑mediated depletion of Vangl1 led to a marked reduction in the number of invading cells, compared with siScramble controls, which displayed dense invasion through the matrix (Fig 7a). Quantification confirmed a statistically significant decrease in invasion upon Vangl1 knockdown, indicating that Vangl1 is required for efficient invasion in this context (Fig 7b).

To further assess the role of Vangl1 in a physiologically relevant 3D environment, we examined spheroid behaviour across increasing collagen content to assess invasiveness under conditions that model mechanical stiffness of the tumour microenvironment. Studies have shown that tumours remodel the extracellular matrix to increase collagen density and stiffness, features that potentiate invasiveness and metastasis [48–51]. So, in addition to assessing invasion, we also deduced whether Vangl1 is required for stiffness‑dependent invasive capacity. We found that control spheroids (siScramble) exhibited clear invasive protrusions, particularly in the 1:1 Matrigel:Collagen matrix at Day 3, where cohesive, finger‑like extensions radiated from an intact spheroid core – hallmarks of organized collective invasion [52,53] (Fig 7c). These protrusions expanded by Day 7, accompanied by sheet‑like spreading highlighted by intensely diffused colour change in the 3D surface plots. In the 1:3 matrix, control spheroids underwent complete dissolution of the spheroidal core and displayed pronounced sheet‑like invasion at both timepoints, suggesting that increased matrix stiffness enhances the aggressiveness and collective invasiveness of CaSki cells.

In contrast, neither E6/E7d‑epleted nor Vangl1‑depleted spheroids generated comparable protrusive structures under any matrix condition. E6/E7 knockdown completely inhibited invasiveness of the spheroids, alluding to the importance of the expression of the oncoproteins in maintaining cervical tumour aggressiveness (Fig 7d). Vangl1‑depleted spheroids displayed significantly fewer invasive protrusions, with the most visible extensions occurring in the 1:3 matrix (Fig 7e). In addition to the loss of invasiveness, Vangl1 depletion produced distinct, matrix‑dependent structural defects and invasion pattern. In the 1:1 matrix, where control spheroids initiated collective invasion, siVangl1 spheroids instead showed shape distension and short, blunt tail-like extensions, usually indicative of compromised epithelial organization, changes in matrix architecture, nuclear rigidity or metabolic stress [54,55]. In the stiffer 1:3 matrix, the spheroids appeared more compact but exhibited scalloped edges and a striking mono‑directional invasion rather than the radial features seen in controls. These suggest that the underlying architectural instability persists but is partially masked by the mechanical constraints of the collagen‑rich environment. Taken together, these data show that Vangl1 is required for maintaining a stiffness-dependent collective invasiveness in transformed cervical cancer cells.

Having established that Vangl1 is required for efficient invasion, we next examined whether AP1M1 contributes to the invasive behaviour of transformed cervical cancer cells. Using the 2D Matrigel invasion assay, siScramble cells displayed robust invasion, as previously seen (Fig 7f). Also consistent with our earlier findings is that Vangl1 depletion markedly reduced invasion, resulting in sparse distributions of invading cells. Notably, AP1M1 knockdown produced a similarly pronounced reduction in invasion, with even lower cell numbers than those observed in siVangl1 condition. This phenotypic overlap indicates that the E7-AP1M1-Vangl1 regulatory relationship is essential for invasive capacity in transformed cervical cancer cells.

Collectively, these observations demonstrate that E7 modulation of Vangl1 is required to maintain the invasive behaviour of transformed cervical cancer cells across diverse extracellular matrix contexts.

### Vangl1 distribution is enriched at invasive fronts of CaSki spheroids

**Fig 8:**
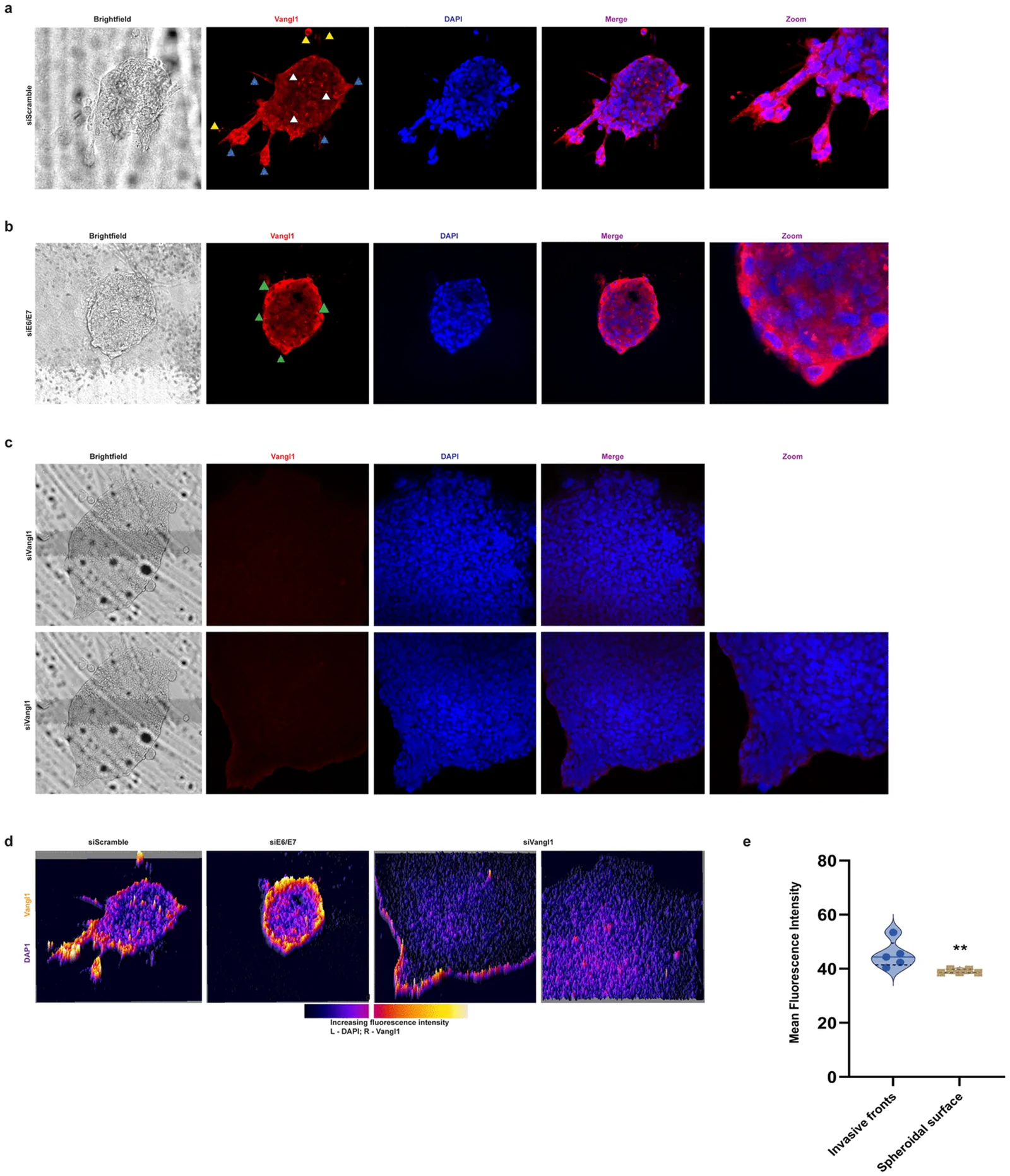
Vangl1 is enriched at the leading-edge of invading protrusions of CaSki spheroids. **(a)** Immunofluorescence images of collectively invading siScramble (control) CaSki spheroids embedded in a 1:1 Matrigel:Collagen matrix. Confocal microscopy at 40 × magnification reveals strong Vangl1 enrichment at the outer edge of leading cells within invasive protrusions. The side zoom panel highlights Vangl1 accumulation at the advancing front of the protruding cell clusters. **(b)** Immunofluorescence images of siE6/E7 CaSki spheroids showing a non‑invading phenotype. Vangl1 displays a restored cortical distribution along the spheroid perimeter, consistent with restoration of Vangl1 localisation. This organized cortical pattern is further emphasized in the zoom panel. **(c)** Immunofluorescence images of siVangl1 CaSki spheroids showing markedly reduced Vangl1 signal with significantly reduced leading‑edge enrichment compared with the control spheroids. Loss of Vangl1 results in increased cellular packing density and a corresponding increase in spheroid volume. Due to this enlarged, densely packed morphology, the complete spheroid could not be captured within a single microscope frame and was therefore imaged in halves. Corresponding brightfield images were acquired at 20 × magnification. Cell nuclei were stained with Hoeschst 33342, and endogenous anti-Vangl1 antibody was counterstained with anti-mouse Alexa Fluor 594. Micrographs were prepared using ImageJ and the brightness of the panels was uniformly enhanced post image processing. **(d)** 3D surface plots of spheroids immunofluorescence show regions of Vangl1 accumulation as increasing colour intensities of purple to yellow peaks. DAPI nuclear staining appears as blue/purple-coloured peaks. **(e)** Fluorescence intensity of Vangl1 signal at the invading leader cells and the spheroidal surface of siScramble spheroids were generated using ImageJ. The mean gray value was used to plot the mean fluorescence intensity. The representative sections measured are shown in S3 Fig.

Having established that Vangl1 is essential for collective invasion phenotypes in CaSki spheroids, we then examined how HPV-driven transformation influences Vangl1 distribution and whether this creates polarity cues that support this invasive phenotype. We examined Vangl1 subcellular localization in CaSki spheroids by immunofluorescence in invading spheroids embedded in 1:1 Matrigel:Collagen matrix following siRNA transfection with siScramble (control), siE6/E7 or siVangl1. As shown in Fig 8a-e, immunofluorescence staining revealed marked differences in Vangl1 organization under the different conditions. In siScramble cells (Fig 8a), which represent the control, Vangl1 displayed a disorganized and heterogeneous pattern. The signal was distributed across diffuse cytoplasmic regions without consistent cortical enrichment, similar to the observations in HEK293 cells co-expressing HPV-16 E7 and Vangl1 (Fig 6a). The diffused cytoplasmic signal was interspersed with irregular, fragmented puncta (white arrows). Notably, Vangl1 appears to localize preferentially to invading sites along the spheroidal edge. Also, the outer edge of leading cells (blue arrows) displayed strong enrichment of Vangl1, forming continuous arcs at the migration front of collectively invading cells. This is corroborated by the deduced relative fluorescence intensity which reveals that the intensity of Vangl1 around the leader cells is significantly and several folds higher than at the spheroidal surface. (Fig 8e; Fig S4). This localization coincided with elongated, directionally oriented clusters of cells, indicating a role of Vangl1 in the front–rear cell coordination required for collective movement. In addition, single disseminated cells (yellow arrows) were also seen, suggesting a multimodal invasive capacity of the spheroids. Collective migration is marked by the transition of a static, apico-basally polarized epithelial cell into a motile, front-rear polarized cell capable of movement. While the apico-basal polarity is lost during this transition, several studies have shown that the PCP signalling remains functionally active and redistributed along the migration axis, contributing to polarized activities at the leading edge and at the rear [56–59]. Consistent with this, previous reports show that the localization of PCP proteins such as Vangl or Frizzled to the leader cells is a highly significant pro-migratory attribute [24,60]. Therefore, our observations of Vangl1 enrichment in leader cells of collectively invading cervical cancer cells suggest that Vangl1 is spatially repurposed to enhance collective invasiveness.

Strikingly, transient depletion of E6/E7 partially restored Vangl1 organization (Fig 8b). Compared with siScramble cells, siE6/E7 cells exhibited a more structured Vangl1 signal especially on the spheroidal edge and formed fewer and smaller puncta. In addition, the cells showed increased cytoplasmic and cortical accumulation and localisation of Vangl1. These changes suggest that repression of E6/E7 rescues aspects of planar polarity and vesicular trafficking, allowing Vangl1 to adopt a more physiologically ordered distribution. As expected, siVangl1 treatment markedly reduced Vangl1 signal (Fig 8c). However, the residual Vangl1 signal remains heterogeneous and diffuse, resembling that of the siScramble spheroids but with markedly reduced accumulation around the weakly-invading cell clusters that formed tail‑like extensions. The DAPI signal further reveals that siVangl1 spheroids contain tightly packed nuclei, indicating increased cellular packing density and an overall increase in spheroid volume. This phenotype aligns with the inverse relationship between invasion and spheroid volume that occurs in 3D tumour models [51,54]. Highly invasive spheroids disperse cells into the surrounding matrix, relieving internal crowding and mechanical stress, as seen in the siScramble condition (Fig 8a). In contrast, when invasion is impaired, as in siVangl1 spheroids (Fig 8c), cells remain confined within the aggregate, leading to increased compressive stress [55,61,62]. Although this can appear as enhanced proliferation, the resulting increase in compressive stress restricts cell‑cycle progression and promotes apoptosis, ultimately increasing necrosis within the spheroid core [61–64]. It is important to note that while the siE6/E7 spheroids also lost invasive capacity, there was no increase in cellular packing density as seen in siVangl1. This, further emphasises that, while phenotypes of siVangl1 may mirror siE6/E7 spheroids in certain responses, Vangl1 plays a unique role that directly impacts cellular architecture and organization in cervical cancer tumours.

To further define the spatial organization of Vangl1 in these spheroids, we used 3D surface plots to generate volumetric fluorescence reconstructions of the 2D immunofluorescence images in Fig 8a-c. In siScramble spheroids, Vangl1 (bright yellow colour) accumulated as intense peaks strictly at the invasive edges and formed distinctively high‑intensity ridges in invading leader cells (Fig 8d-e; S4 Fig). There were also sparse peaks of Vangl1 corresponding to fragmented puncta across the spheroidal surface (red peaks). In contrast, siE6/E7 spheroids show a defined circumferential distribution of high-intensity ridges of Vangl1. In addition, Vangl1 signal increased across the spheroidal surface compared with siScramble spheroids. Lastly, siVangl1 spheroids revealed dense nuclear packing appearing as multi‑layered DAPI (blue/purple coloured) peaks across the spheroidal surface, with only short peripheral Vangl1 peaks.

Taken together, these findings indicate that Vangl1 is required not only for maintaining epithelial architecture, but also for positioning polarity machinery at the migration front to allow cohesive, collective invasion of transformed cervical cancer cells. Furthermore, the findings suggest that Vangl1 contributes to the maintenance of the mechanical cues in the cervical tumour microenvironment needed for tumour progression and metastasis.

### Vangl1 depletion enhances chemosensitivity in 2D and 3D CaSki models

Given the identified oncogenic contributions of Vangl1 and the emerging role of polarity regulators in therapy response, we investigated whether Vangl1 contributes to chemoresistance in HPV-positive cervical cancer cells. Polarity disruption is increasingly recognised as a mechanism by which tumour cells evade immune surveillance and withstand cytotoxic stress [39], and the loss or mislocalization of polarity proteins such as Scribble have been linked to cisplatin resistance in multiple cancers [65,66]. We therefore assessed how depletion of Vangl1 influences chemosensitivity in CaSki cells.

**Fig 9:**
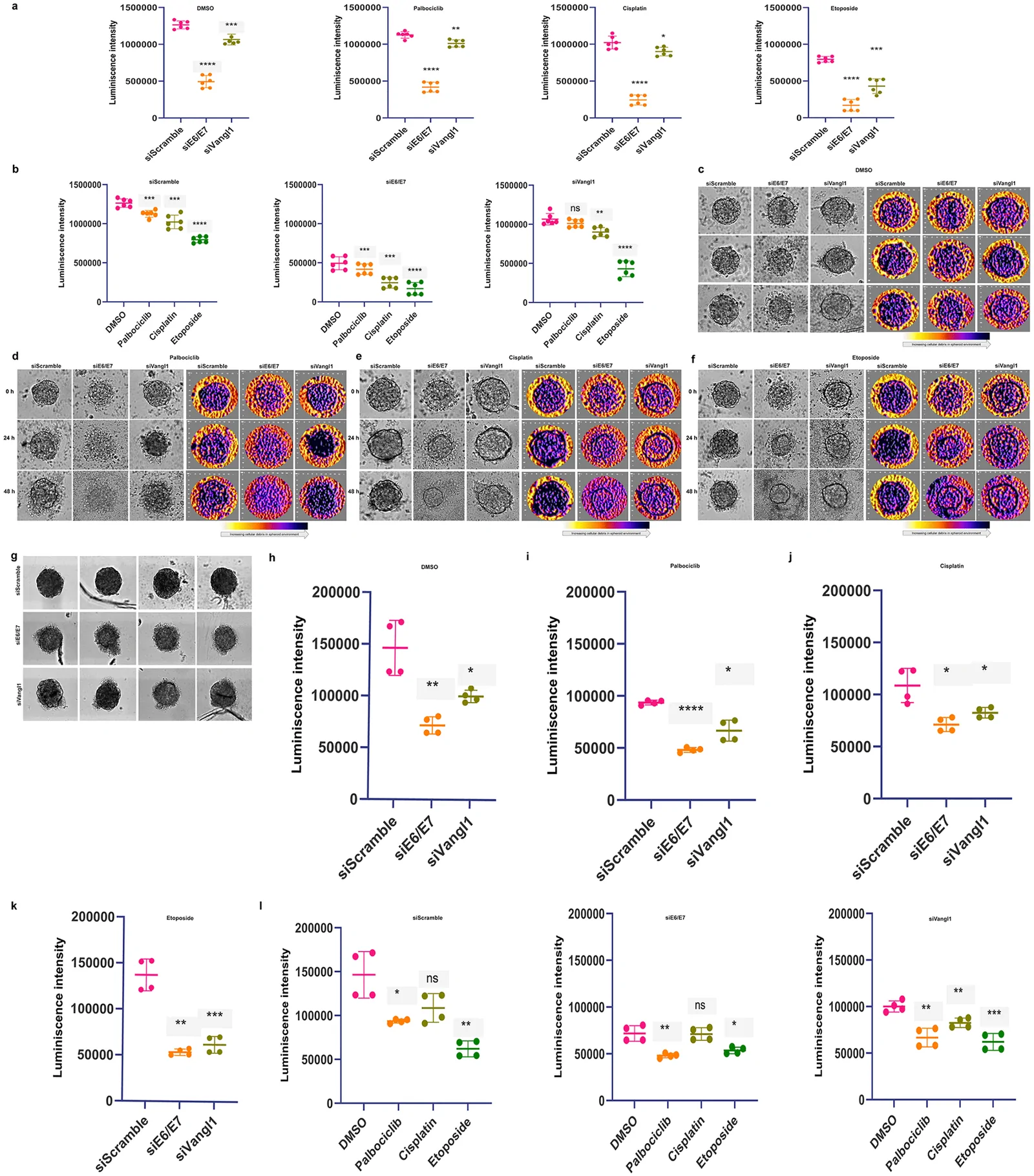
Loss of Vangl1 enhances the chemosensitivity of cervical cancer cells. (a–b) Cell viability of monolayer CaSki cells following siRNA-mediated depletion of E6/E7 or Vangl1 and subsequent treatment with DMSO, Palbociclib, Cisplatin, or Etoposide. Data represents means of luminescence intensities of six biological replicates ± standard deviation. (c–f) Brightfield images of CaSki spheroids 48 h after siRNA transfection and an additional 48 h of treatment with the indicated chemotherapeutics. The corresponding 3D surface plots depict the spatial distribution of cells within the spheroid environment, where the shift in colour thermal intensity reflects increasing levels of cell dispersal or debris accumulation. Brightfield images were acquired at 20 × magnification. Micrographs and 3D surface plots were processed using ImageJ, and brightness was uniformly enhanced across panels. (g) Brightfield images of representative CaSki spheroids for the viability assays (h–l) Cell viability of CaSki spheroids following chemotherapeutic treatment. Data represents means of luminescence intensities of four biological replicates ± standard deviation. *p* values of 3 independent experiments were calculated by Student’s t test. *: *p*-value < 0.05; **: *p*-value <0.01; ***: *p*-value <0.001; ****: *p*-value <0.0001.

To do this, we assessed cell viability in response to DMSO, Palbociclib, Cisplatin, or Etoposide, in 2D monolayer and 3D spheroid cultures of CaSki cells. In monolayer cultures, siRNA-mediated depletion of E6/E7 or Vangl1 significantly reduced baseline viability even under DMSO treatment, confirming that both targets are required for optimal survival of transformed CaSki cells (Fig 9a&b). Upon treatment with Palbociclib, Cisplatin, or Etoposide, siE6/E7 and siVangl1 cells exhibited markedly reduced viability compared with siScramble controls. The strongest differential response was observed with Etoposide, whereas Palbociclib produced the mildest effect. Notably, siVangl1 cells displayed enhanced sensitivity to Cisplatin and Etoposide, while their response to Palbociclib was less pronounced (Fig 9a&b). These findings indicate that depletion of Vangl1, similar to loss of E6/E7, primes CaSki cells for treatment-induced loss of viability in 2D.

To further evaluate whether Vangl1 influences drug response, we next assessed chemotherapeutic sensitivity in CaSki spheroids, which better recapitulate tumour architecture and spatial polarity. Forty-eight hours after siRNA transfection (recorded as 0-h of treatment), spheroids were treated with the same drug panel as the monolayer culture and monitored for morphological changes and ATP-based viability. Morphological alterations of 3D models are crucial and highly reliable readouts of chemosensitivity. These include size reduction, spheroid dissociation or disintegration, surface or edge irregularity [67,68]. Under DMSO treatment, siScramble spheroids remained compact and cohesive, whereas siE6/E7 and siVangl1 depleted spheroids progressively lost structural integrity, exhibiting edge-fraying, reduced height, and diminished central density (Fig 9c). This baseline structural instability induced by the respective ablations was amplified under drug treatment. Palbociclib induced mild disintegration in siScramble spheroids but caused pronounced dissociation in siE6/E7 and siVangl1 spheroids (Fig 9d). Cisplatin (Fig 9e) and Etoposide (Fig 9f) produced even stronger effects: siScramble spheroids showed moderate disruption, while siE6/E7 and siVangl1 spheroids displayed fragmented contours, extensive cell shedding, reduced spheroid size, and a halo of debris (Fig 9c). While Palbociclib largely disrupted the compactness of the spheroids, Cisplatin and Etoposide caused peripheral disintegration and reduction in spheroid sizes. The corresponding 3D surface plots depict the spatial distribution of cells within the spheroid environment, where the shift in thermal colour intensity from yellow to purple reflects increasing levels of cell dispersal or debris accumulation.

In a similar setup, we evaluated cell viability by measuring ATP levels in CaSki spheroids after 48 h of drug treatment (Fig 9g-l). Viability assays revealed a notable divergence in Cisplatin response. Unlike in 2D, both siScramble and siE6/E7 spheroids exhibited relative Cisplatin resistance, consistent with the known protective architecture of 3D tumour models. Strikingly, Vangl1 depletion overcame this resistance, rendering spheroids significantly more sensitive to Cisplatin. Moreover, in contrast to 2D cultures, siVangl1 spheroids were also highly sensitive to Palbociclib, with a response magnitude comparable to Etoposide.

Collectively, these findings demonstrate that depletion of E6/E7 or Vangl1 sensitizes CaSki cells to chemotherapeutics, but the magnitude and pattern of response differ between 2D and 3D contexts. Importantly, Vangl1 loss uniquely enhances cisplatin-sensitivity in spheroids, revealing a polarity‑dependent mechanism of chemoresistance that is not captured in 2D cultures. These results position Vangl1 as a polarity‑linked determinant of therapeutic response in HPV‑positive cervical cancer.

### Vangl1 knockdown markedly reduced Dvl2 protein levels

**Fig 10:**
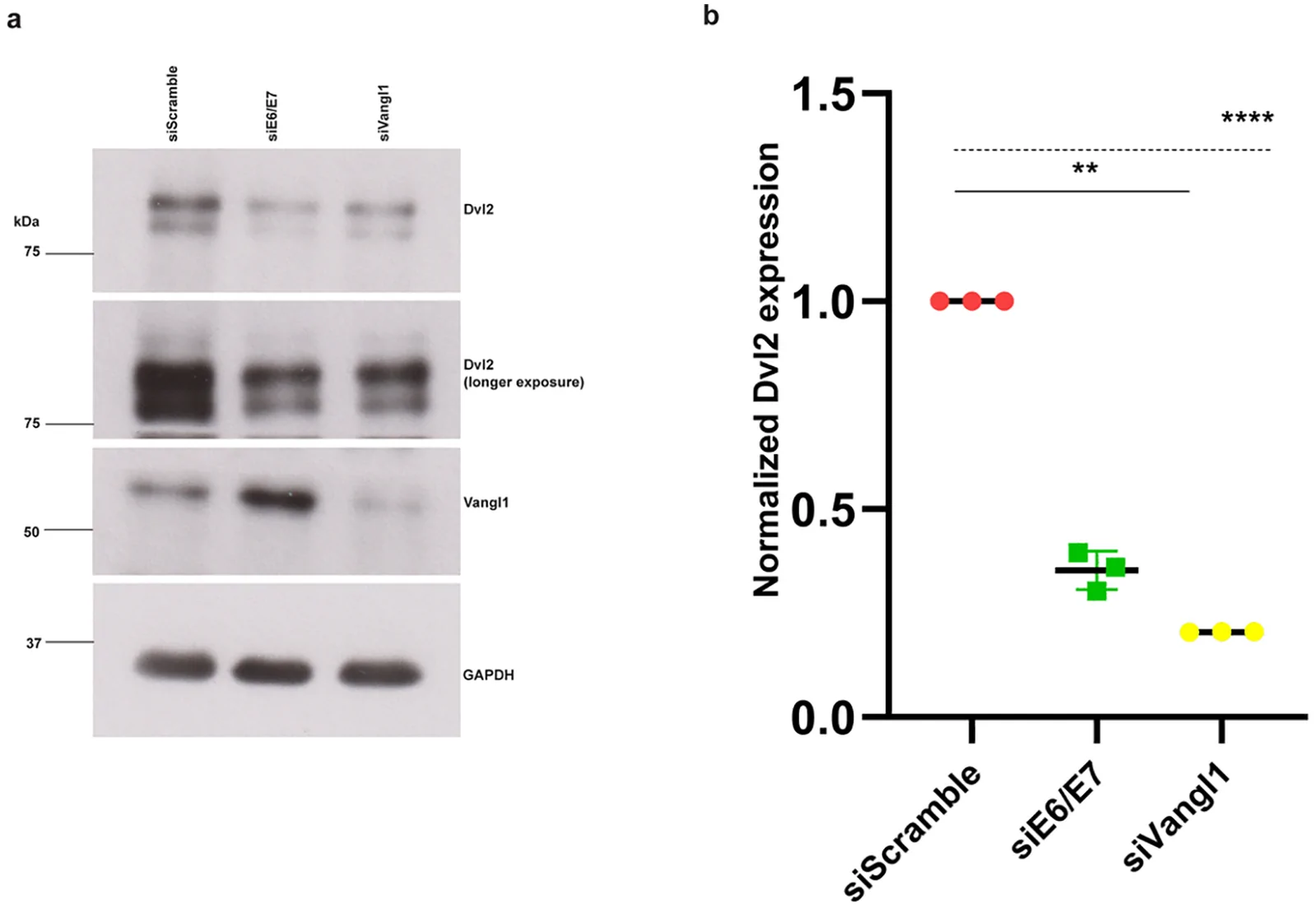
Dvl2 is elevated in CaSki cells. (a) Western blot analyses of CaSki whole cell lysates following siRNA transfection. The knockdown of E6/E7 or Vangl1 significantly reduces Dvl2 protein levels. Dvl2 band density was normalized to GAPDH band density, and the data was used to plot the densitometry graph of relative Dvl2 expression. Data is shown as the fold-changes of normalized Dvl2 levels relative to the control (siScramble) ± standard deviation. *p* values of 3 independent experiments were calculated by Student’s t test*: *p*-value < 0.05; **: *p*-value <0.01; ***: *p*-value <0.001).

We further sought to delineate the role of Vangl1 in potentiating an E7-mediated PCP dysregulation by assessing other components of the Wnt/PCP. In the PCP pathway, a mutual antagonism is required between Frizzled/Dvl and Vangl/Prickle complexes for efficient polarisation [21,69]. The Vangl/Prickle complex inhibits the downstream activation of Dvl-RhoA-Rock1 complex responsible for cytoskeletal rearrangements and migration [70,71]. In particular, elevated Dvl2 levels, due to its activation, have been implicated in PCP-mediated metastasis across different cancers [47,72]. Therefore, we investigated Dvl2 dynamics and the downstream effect of Vangl1 dysregulation in cervical cancer cells. Our preliminary investigation shows that CaSki cells express elevated levels of Dvl2, which is significantly reduced upon knockdown of E6/E7 or Vangl1 (Fig 10a and b). While further investigations into Dvl2 modulation and other PCP components are ongoing, our preliminary findings suggest that the interaction of Vangl1 and E7 contributes to elevation of Dvl2 levels, which may, in turn, enhance invasion.

## Discussion

High-risk HPV E7 proteins classically target the pRb tumour suppressor, but continuing research shows that E7 also influences cellular pathways beyond cell cycle control. In this study, we identify Vangl1, a core component of the Wnt/planar cell polarity (PCP) pathway [23], as a novel, phosphorylation-dependent interactor of HPV-16 E7. Overexpression or loss of Vangl1 has been reported to promote malignancy in breast cancer, glioblastoma multiforme, and lung adenocarcinoma [24,27,46,73]. Thus, the fate and role of Vangl1 in cancers is complex and context-dependent. This study therefore provides new insights into HPV-mediated cervical cancer by linking the E7 oncoprotein to a signalling axis that controls planar polarity and epithelial organization.

Our findings demonstrate that CKII phosphorylation of the HPV-16 E7 CR2 region is essential for Vangl1 binding. We found that Vangl1 binds strongly to phosphorylated HPV-16 E7, weakly to phosphorylated HPV-18 E7, and shows no detectable binding to the low-risk or cutaneous HPV E7 proteins examined. This specificity mirrors the oncogenic potential of these HPV subtypes [5] and suggests that recruitment of Vangl1 by HPV-16 may contribute to its pathogenicity. Additionally, the inability of phosphomimic peptides to recapitulate Vangl1-E7 binding further indicates that the interaction depends on actual structural features conferred by true phosphorylation rather than charge alone. CKII-mediated phosphorylation of HPV-16 E7 at serine residues 31 and 32 (or 33 in HPV-18 E7) influences its interaction with known substrates like pRb, amplifying its degradation and consequently, cell transformability [15]. Therefore, these observations establish a crucial biochemical requirement for Vangl1 recruitment and reiterate the importance of CKII phosphorylation on E7’s interactome.

Furthermore, our findings demonstrate that HPV-16 E7 specifically binds Vangl1 but not Vangl2. This confirms that Vangl1 can be regulated differently from Vangl2, despite their close structural similarities [74]. A key insight from this study is that E7 not only binds Vangl1 but also modulates its abundance and turnover in HPV-transformed cervical cancer cells. Interestingly, this oncoprotein-specific modulation of Vangl1 protein is not fully explained by inhibition of canonical degradation pathways. Rather, cycloheximide chase assays showed a distinct biphasic post-translational decay of different Vangl1 forms, whereby unphosphorylated Vangl1 is rapidly degraded while there is an aberrant stabilization of the phosphorylated form. In addition, this prolonged retention of Vangl1 led to stabilization of E7. This bidirectional stabilization suggests that E7 contributes to the persistence of Vangl1 to, potentially, establish an oncogenic signalling. Studies have shown that high-risk E7 oncoprotein modulates several host factors for its own (e.g. pRB, USP7, USP11), or its substrate’s stabilization (e.g., APOBEC3As) [75–78]. These regulations occurring through direct interaction, inhibiting degradation, or through a recently described chaperone activity (with pRB), contribute towards establishing or sustaining malignancy in a cervical cancer context.

Mechanistically, our study indicates that E7 alters Vangl1 trafficking through the clathrin adaptor subunit AP1M1. In normal cells, AP1M1 is responsible for the endocytic sorting of Vangl1, facilitating its export from the TGN to the cell cortex, where it assembles into PCP complexes and interacts with cytoskeletal regulators [36]. Therefore, sequestering AP1M1 can impair Vangl1 localization and function. We found that AP1M1 binds phosphorylated HPV-16 E7, and the effects of its depletion resemble those of E6/E7 knockdown, by increasing Vangl1 abundance and reducing E7 levels. Furthermore, we found that E7 influences Vangl1 localization, disrupting its distribution to the cytoskeletal compartments. Since Vangl1’s function as a PCP scaffold depends on its precise spatial distribution, E7-driven mislocalization disrupts polarity cues and contributes to the altered epithelial architecture characteristic of HPV-transformed cells. In addition, these observations further clarify the non-canonical Vangl1 rescue upon E7 depletion, as well as the prolonged half-life of Vangl1, as possible consequences of mislocalization. This is because altered localization can sequester proteins away from normal turnover and affect overall protein abundance [32,33]. This study also supports previous reports that CKII-phosphorylated E7 contributes to the mislocalization of PDZ-containing polarity proteins that are primary targets of the E6 oncoprotein [17,20].

The functional consequences of this regulatory network are seen in CaSki spheroid models, where Vangl1 depletion modestly phenocopies E6/E7 loss by inducing spheroid disintegration and loss of structural cohesion. These parallel phenotypes indicate that Vangl1 is not merely a passive target of E7, but an active effector required for maintaining epithelial integrity in HPV-positive cells. Beyond its role in maintaining spheroid architecture, Vangl1 is essential for the invasive capacity of transformed cervical cancer cells. Its loss produces distinct, quantifiable compromises in both architecture and invasive capacity, with the most pronounced defects emerging under conditions that normally support organized collective invasion. Indeed, Vangl1 knockdown markedly impaired invasion in both 2D and 3D models, underlining its requirement for efficient matrix engagement and directional movement. Of particular significance, Vangl1 depletion abolished the hallmark collective invasion phenotype exhibited by wild‑type CaSki spheroids.

Although metastasis has traditionally been viewed as a predominantly single‑cell process driven by loss of adhesion, accumulating evidence shows that tumour cells can migrate and disseminate as cohesive clusters that retain cell–cell contacts. Such collective invasion is directed by leader cells that extend into the surrounding matrix while remaining mechanically coupled to follower cells, generating contiguous invasive strands [52]. This behaviour closely mirrors the invasive phenotype of wild‑type CaSki spheroids in our study. Consistent with establishing the front-rear polarity cues required for this process [60,79], we observed striking enrichment of Vangl1 at the leading edge of invasive protrusions, positioning it at the site where directional and collective coordination are established. These findings reinforce the idea that Vangl1 is a critical determinant of collective motility and a crucial effector through which E7 reshapes planar polarity and stabilizes oncogenic architecture in transformed cervical contexts.

In addition to its role in sustaining epithelial architecture and collective invasion, Vangl1 also proved essential for the ability of cervical cancer spheroids to withstand therapeutic stress. In response to treatment with all drugs (Etoposide, Cisplatin, and Palbociclib), the most severe structural deterioration and loss of viability occurred in spheroids lacking E6/E7. This reinforces the requirement for the continuous expression of the oncoproteins in sustaining the viability of cervical cancer cells [4,7]. And that repressing the expression of these oncoproteins is a core vulnerability that enhances susceptibility of cervical cancer cells to chemotherapeutics. The modestly comparable morphological and cytotoxic effects seen upon Vangl1 depletion also indicate its contribution to maintaining viability under genotoxic and cytostatic stress. This supports the notion that Vangl1 dysregulation creates a downstream effector through which E7 maintains the structural resilience of transformed epithelial architecture. Moreover, Vangl1 depletion enhanced the cells’ chemosensitivity to drugs with distinct mechanisms of action, suggesting that Vangl1 dysregulation contributes broadly to the cytostatic and genotoxic stress tolerance of cervical cancer cells. Our study therefore positions the Wnt/PCP pathway as a promising therapeutic axis for HPV-associated cervical cancer by identifying Vangl1-PCP signalling as a viable druggable target.

To further contextualize the role of HPV‑16 E7 within the PCP network, we examined whether additional PCP components are altered in HPV‑positive cells, especially through the involvement of Vangl1. As a first step, we assessed Dvl2, a key protein that serves as a central switch in non-canonical Wnt signalling pathways, influencing cell migration and cytoskeletal rearrangements [47,70–72]. In the PCP, Dvl activation is tightly regulated by its inhibition by the Vangl-Prickle complex, hence, any perturbations to the homeostasis of this interaction could cause its aberrant expression. Invasion in various cancers, including breast cancer, pancreatic cancer, and colorectal cancer, has been linked to Dvl2 activation, which has, in some cases, proven to be Vangl-mediated [71]. Our preliminary analyses indicate that Dvl2 protein may be activated in CaSki cells as E6/E7 knockdown drastically reduces its levels (Fig 10a and b). Interestingly, the knockdown of Vangl1 also decreased Dvl2, even lower than E6/E7 knockdown. This suggests that Dvl2 activation in CaSki cells might be linked to Vangl1 dysregulation. These early findings support the possibility that E7’s effects on the PCP may extend beyond its modulation of Vangl1 and may influence additional nodes.

In conclusion, our study identifies a previously unrecognized role for HPV‑16 E7 in modulating planar cell polarity signalling and positions Vangl1 as a phosphorylation‑dependent substrate contributing to HPV‑driven epithelial disorganization. Although Vangl1 has been implicated in a few non‑PCP contexts, the behaviour we observe here aligns strongly with classical PCP‑associated activities, including changes in collective migration and protrusive leader cell dynamics. While our findings point toward PCP‑tilted mechanisms, we acknowledge that this work does not yet provide a full spatial map of all core PCP proteins in relation to E7 expression. Ongoing studies are now focused on visualizing their asymmetric localization to strengthen this mechanistic link.

We also note that although E6/E7 and Vangl1 knockdown produce similar disruptions in cell behaviour, the more pronounced phenotypes following E6/E7 ablation likely reflect apoptosis triggered by loss of the viral oncoproteins. Future work will explore how Vangl1 mislocalization influences downstream PCP effectors, how endocytic trafficking contributes to these changes, and how these pathways collectively shape early cervical transformation in the HPV context. Lastly, a deeper understanding of how HPV manipulates planar polarity signalling may reveal further new therapeutic targets.

## Materials and methods

### Cell culture and reagents

HEK‑293 cells, normal human keratinocytes, HaCaT cells, HPV‑16/18–positive cervical cancer cell lines (CaSki and HeLa), and the HPV‑negative cervical cancer line C‑33A were obtained from ATCC. Cells were maintained in DMEM (Gibco, 31885‑023) supplemented with 10% FBS (Gibco, 10270‑106), penicillin–streptomycin (100 U mL^−1^), and glutamine (300 μg mL^−1^; Gibco, 10378‑016). Cultures were grown at 37°C in a humidified incubator with 10% CO_2_.

### Antibodies, inhibitors, antibiotics and chemotherapeutics

Primary antibodies included anti‑Vangl1 (Merck, AMAB90600), anti‑RB1/pRB (BD Pharmingen, 554136), anti‑FLAG (Merck, F7425), anti‑HA (Abcam, ab9110), anti‑HPV‑16 E7 (SCBT, sc65711), anti‑phospho‑HPV‑16 E7 was generated by Eurogentec as previously described (16), anti‑HPV‑18 E7 (SCBT, sc-365035), anti-AP1M1 (Merck, SAB1301057), anti-Dvl2 (SCBT, sc-8026), anti‑GAPDH (SCBT, sc-32233), anti‑β‑galactosidase (Promega, Z378B), anti-EGFR (SCBT, sc-373746), anti-Vimentin (SCBT, sc-6260), anti-Vinculin (CST, 4650T), HRP‑conjugated anti‑mouse (115-035-071) and anti‑rabbit (111-035-046) secondary antibodies were from Jackson ImmunoResearch.

MG132 (Sigma‑Aldrich, C2211) was used for proteasome inhibition, cycloheximide (Sigma‑Aldrich, C4859) for protein synthesis inhibition, and chloroquine phosphate (Sigma‑Aldrich, PHR1258) for lysosomal inhibition. CX-4945 (Silmitasertib) was used for inhibiting casein kinase II phosphorylation. Ampicillin (Merck, A5354) was used in bacterial cultures for selection.

Etoposide (Merck, E1383), Cisplatin (Merck, C2210000) and Palbociclib (CST, 47284) were used as chemotherapeutics.

### Plasmid constructs and transfections

pGEX‑2T constructs encoding HPV‑5 E7, HPV‑11 E7, HPV‑16 E7, and HPV‑16 E7 C‑terminal fragments were generated as described [80]. Wild‑type pGEX‑18E7 and FLAG/HA‑tagged pCMV16 E7 plasmids [81] were gifts from Karl Münger. E7 fragments were subcloned into pCMV GFP plasmid, a kind gift from Connie Cepko (Addgene,11153). The CKII α/β expression vector (pCDF CKII duet) was provided by Yong Xiong and John Guatelli. FLAG‑Vangl1 was provided by Pablo Rodriguez‑Viciana [82] and Vangl2 ORF was cloned into pcDNA3.1 (Genscript). All constructs were sequence‑verified.

HEK‑293 cells were transfected using the calcium phosphate precipitation method [83].

### Peptide synthesis

Peptides were synthesized at the ICGEB Biotechnology Development Unit. Scrambled peptides contained 10 random amino acids. Non‑phosphorylated peptides corresponded to HPV‑16 E7 CR2 (residues 15–39; wild‑type or N29S). Phosphorylated peptides contained serine phosphorylation at S29, S31, and S32.

### Peptide pulldown assay

HaCaT cells (80–90% confluent) were lysed in RIPA buffer containing 50 mM Tris‑Cl pH 7.4, 150 mM NaCl, 1% NP‑40, 0.5% sodium deoxycholate, 0.1% SDS, and protease inhibitors. Lysates were clarified by centrifugation (16,000 rpm, 10 min, 4°C). Biotinylated peptides (500 μg) were bound to streptavidin magnetic beads (GE Healthcare) for 1 h at 4°C. Pre‑cleared lysates were incubated with peptide‑bound beads for 2 h at 4°C. After extensive washing, 5% of the beads were analyzed by Western blotting; the remainder was digested with trypsin and analyzed by mass spectrometry.

### Mass spectrometry

Protein complexes were analyzed as described previously [84]. Samples were loaded onto Picofrit nanobore columns packed with 1.8 μm Zorbax XDB C18 resin and analyzed on an LTQ ion trap mass spectrometer (Thermo Electron). One full MS scan (400–1700 m/z) was followed by eight data‑dependent MS/MS scans. RAW files were converted to mzXML (READW v1.6) and searched against Ensembl human and NCBInr viral databases using the Global Proteome Machine with X!Tandem.

### GST‑fusion protein purification

pGEX‑2T constructs (GST empty, GST‑5E7, GST‑11E7, GST‑16E7, GST‑16E7 C‑T, GST‑18E7) were transformed into *E. coli* BL21(DE3) pLysS. For phosphorylated GST‑E7 proteins, constructs were co‑transformed with pCDF‑CKII. Cultures were induced with 200 μM IPTG at 25°C for 16–20 h. Bacteria were lysed in PBS with 1% Triton X‑100, sonicated, clarified, and incubated with glutathione agarose beads (Sigma, G4510). Beads were washed and stored in PBS with 1% Triton X‑100 and 30% glycerol at −20°C.

### GST pull‑down assay

HaCaT cells were lysed in RIPA buffer on ice for 10 min and clarified by centrifugation. Lysates were incubated with GST‑fusion proteins for 2 h at 4°C. Beads were washed with E1A buffer (50 mM HEPES pH 7.0, 250 mM NaCl, 0.1% NP‑40), eluted in 2 × SDS loading buffer, and analyzed by SDS‑PAGE and Western blotting.

### Co‑immunoprecipitation

HEK293 cells were transfected as indicated and lysed in RIPA buffer after 18 h. Clarified lysates were incubated with EZview Red Anti‑HA Affinity Gel (Sigma, E6779) for 4 h at 4°C. Beads were washed, eluted in 2 × SDS loading buffer, and analyzed by SDS‑PAGE and Western blotting.

### siRNA transfection

CaSki, HeLa, C‑33A and HaCaT cells were transfected with siRNAs targeting Vangl1 (Dharmacon SMARTpool), AP1M1 (Dharmacon SMARTpool), HPV‑16 E6/E7 (Eurofins), or Scramble control (Dharmacon siSTABLE) as previously described [78]. Cells were harvested after 72 h for Western blotting or downstream assays.

For spheroids, siRNA mixes were prepared in serum‑free DMEM, but spheroids were maintained in DMEM with FBS and PSG. Partial media replacement was performed after 24 h to preserve spheroid integrity.

### Western blotting

Samples were denatured at 95°C for 10 min, separated by SDS‑PAGE, and transferred to 0.22‑µm nitrocellulose membranes. Membranes were blocked in 5% milk/TBS‑T for 1 h, incubated with primary and secondary antibodies, and developed using ECL (GE Healthcare, RPN2106).

### Proteasomal and lysosomal inhibition

Cells transfected with siRNAs were treated with 20 µM MG132 or chloroquine for 6 h (endogenous proteins) after 72 h. Then, cells were harvested for Western blot analysis.

### Cycloheximide chase

CaSki cells transfected with siRNA for 72 h were treated with 50 µg/mL cycloheximide and harvested at defined time points (0–10 h). HEK293 cells overexpressing Vangl1, HPV-16 E7, or both were treated similarly, after 24 h of transfection. Protein stability was assessed by Western blotting.

### Lambda (λ) phosphatase treatment

λ‑phosphatase treatment was performed to validate the phosphorylation status of Vangl1. After transfection, cells were lysed in ice‑cold lysis buffer lacking phosphatase inhibitors (50 mM Tris‑HCl pH 7.5, 150 mM NaCl, 1% NP‑40, 1 mM MnCl_2_, and protease inhibitors). For each sample, 20 µg of total protein was incubated with 400 U λ‑phosphatase (New England Biolabs, P0753) in 1 × Protein Metallophosphatases buffer supplemented with 1 mM MnCl_2_ at 30 °C for 45 min. Control reactions were processed in parallel under identical conditions but without enzyme. Reactions were terminated by adding 2 × sample buffer and heating at 95 °C for 5 min. Samples were resolved by SDS‑PAGE and analysed by immunoblotting using anti‑Vangl1 antibodies. Loss of the upper (phosphorylated) Vangl1 band and a shift in gel mobility is interpreted as evidence of Vangl1 dephosphorylation.

### Two‑dimensional Matrigel invasion assay

Matrigel invasion chambers (Corning BioCoat) were rehydrated with serum‑free DMEM for 2 h. siRNA-transfected CaSki cells (5 × 10^4^) were seeded into the upper chamber in serum‑free DMEM; the lower chamber contained DMEM with 10% FBS. After 20 h, invading cells were fixed, permeabilized, stained with 0.05% Crystal Violet, and imaged at 10 × magnification. At least five fields were analyzed per sample.

### Generation of 3D CaSki spheroids

Spheroids were generated by seeding 5,000 CaSki or HaCaT cells in 100 µL per well in ultra-low attachment 96‑well round‑bottom plates (Corning; faCellitate). Plates were incubated at 37°C and 10% CO_2_. Compact spheroids formed within 48 h, with diameters of 160–170 µm. Spheroids were further incubated for 48 h before being used for assays.

### Collagen invasion assay

Rat Collagen I was neutralized to pH 7.0, and Matrigel–collagen mixtures (1:0, 1:1, 1:3) were prepared. Twenty microliters of matrix were dispensed in wells. Spheroids were transferred into the matrix and polymerized for 30 min at 37°C. Wells were filled with DMEM + 5% FBS. Invasion was monitored for 7 days and imaged at 20 × magnification using Nikon Eclipse Ti2 inverted microscope equipped with the NIS-Elements AR 5.02. For immunofluorescence, coverslips were pre-coated with 1 mg/mL poly‑L‑lysine before adding the matrix onto them

### Immunofluorescence

Spheroids embedded in collagen were fixed with 4% paraformaldehyde for 20 min, permeabilized overnight at 4°C in 0.5% Triton X‑100, and blocked in PBS containing 5% horse serum, 0.1% Triton X‑100 for 2 h. Samples were incubated with anti‑Vangl1 overnight at 4°C, washed, incubated with Alexa Fluor 594 secondary antibody for 2 h. Cell nuclei were stained with DAPI and imaged using a Zeiss LSM 880 Airyscan microscope at 40 × magnification. Image analysis was performed using ImageJ v1.54f.

HEK293 cells expressing GFP-16 E7 and FLAG-Vangl1 on poly-L-lysine-coated coverslips were fixed with 4% paraformaldehyde for 10 min, permeabilized for 10 min in 0.1% Triton X‑100, and blocked in PBS containing 1% bovine serum albumin for 30 min. Samples were incubated with anti-FLAG overnight at 4°C, washed, incubated with Alexa Fluor 594 secondary antibody for 1 h. Nuclei staining and imaging were done as mentioned above.

### Chemotherapy treatment of spheroids

CaSki monolayer cells or spheroids pre-transfected with siRNAs for 48 h were treated with 0.1% (v/v) DMSO (as control), cisplatin (10 μM), etoposide (1 μM) or palbociclib (10 μM). Spheroids were imaged at 24 h and 48 h post treatment to assess morphological readouts using Nikon Eclipse Ti2 inverted microscope equipped with the NIS-Elements AR 5.02. In another setup, the CellTiter‑Glo Luminescent Cell Viability Assay (Promega) was used to quantify ATP as a metric index for metabolically active cells in 2D monolayer cultures. After 48 h of chemotherapeutic treatement, we incubated cells with the reagent for 2 min before taking readings. For 3D spheroid cultures, the CellTiter‑Glo 3D assay (Promega) was employed to ensure efficient lysis and ATP extraction from dense multicellular aggregates. After 48 h of treatment, spheroids were incubated with the reagent for 5 mins with agitation to induce lysis before taking readings. Luminescence was recorded using the EnSpire multimode microplate reader, and values were compared to DMSO-treated controls.

### Statistical analysis

Statistical analyses were performed using GraphPad Prism 10. Comparisons between control and siRNA‑treated cells were made using Student’s *t*‑test. Significance was defined as *p* < 0.05. Experiments were performed at least three times, and data are presented as mean ± standard deviation. Significance is indicated as: *p* < 0.05 *, p < 0.005 ***, p < 0.001 ****, and non‑significant (ns) for *p* > 0.05.

Protein quantification was performed using ImageJ v2.16/1.54p, with relative levels calculated as the ratio of target band intensity to loading‑control intensity.

## Supporting information

S1 Fig Western blot of co-immunoprecipitation of FLAG/HA-tagged HPV-16 E7 and FLAG-Vangl1 shows *in vivo* interaction of HPV-16 E7 and Vangl1. Samples were probed with anti-FLAG antibody.
(TIF)

S2 Fig Lambda phosphatase treatment of whole cell lysates showing Vangl1 dynamics in HPV-positive and negative cells. (TIF)

S3 Fig Half-life of total Vangl1 in HEK293 overexpressing Vangl1 only or with HPV-16 E7. Vangl1 band density was normalized to the β-gal band density, and the data was used to plot densitometry graphs of the half-life of total Vangl1.
(TIF)

S4 Fig Fluorescence intensity of Vangl1 signal on siScramble spheroids indicating the enrichment of Vangl1 at invading cell fronts compared with the spheroidal surface and non-invasive fronts. The fluorescence intensity was determined by selecting five representative points (1 –5) (Fig S4a) on each of the spheroidal surface (marked as yellow circles) and the invasive fronts (marked as white circles). The area, mean, intensity density and mean gray values were generated using ImageJ. The background fluorescence was measured by selecting empty regions around the representative points. The obtained values were used to calculate the calculated total cell fluorescence (CTCF) by: CTCF = Integrated Density – (Area of selected cell x Mean fluorescence of background readings). Fig S4b shows the graph of the CTCF indicating that Vangl1 signal is significantly more intense at invasive fronts than at the spheroidal surface.
(TIF)

S5 Fig S1_raw_images. (TIF)

## References

[1] World Health Organization. Cervical cancer. ttps://www.who.int/news-room/fact-sheets/detail/cervical-cancer#:~:text=HPV%20vaccination%20and%20other%20prevention,HPV%20infection%20and%20cervical%20cancer. 2024

[2] Hakim RU, Amin T, Ul Islam SMB. Advances and challenges in cervical cancer: from molecular mechanisms and global epidemiology to innovative therapies and prevention strategies. Cancer Control. 2025;32. doi: 10.1177/10732748251336415 PMC12034968 40267919

[3] Burd EM. Human papillomavirus and cervical cancer. Clin Microbiol Rev. 2003;16(1):1–17. doi: 10.1128/CMR.16.1.1-17.2003 PMC145302 12525422

[4] Jabbar SF, Abrams L, Glick A, Lambert PF. Persistence of high-grade cervical dysplasia and cervical cancer requires the continuous expression of the human papillomavirus type 16 E7 oncogene. Cancer Res. 2009;69(10):4407–14. doi: 10.1158/0008-5472.CAN-09-0023 19435895 PMC3006677

[5] Kurman RJ, Schiffman MH, Lancaster WD, Reid R, Jenson AB, Temple GF, et al. Analysis of individual human papillomavirus types in cervical neoplasia: a possible role for type 18 in rapid progression. Am J Obstet Gynecol. 1988;159(2):293–6. doi: 10.1016/s0002-9378(88)80070-x 2841858

[6] Scheurer ME, Tortolero-Luna G, Guillaud M, Follen M, Chen Z, Dillon LM, et al. Correlation of human papillomavirus type 16 and human papillomavirus type 18 e7 messenger RNA levels with degree of cervical dysplasia. Cancer Epidemiol Biomarkers Prev. 2005;14(8):1948–52. doi: 10.1158/1055-9965.EPI-05-0073 16103442

[7] Jabbar SF, Park S, Schweizer J, Berard-Bergery M, Pitot HC, Lee D, et al. Cervical cancers require the continuous expression of the human papillomavirus type 16 E7 oncoprotein even in the presence of the viral E6 oncoprotein. Cancer Res. 2012;72(16):4008–16. doi: 10.1158/0008-5472.CAN-11-3085 22700879 PMC3421037

[8] Choo CK, Rorke EA, Eckert RL. Differentiation-independent constitutive expression of the human papillomavirus type 16 E6 and E7 oncogenes in the CaSki cervical tumour cell line. J Gen Virol. 1994;75 (Pt 5):1139–47. doi: 10.1099/0022-1317-75-5-1139 8176374

[9] Zhou L, Qiu Q, Zhou Q, Li J, Yu M, Li K, et al. Long-read sequencing unveils high-resolution HPV integration and its oncogenic progression in cervical cancer. Nat Commun. 2022;13(1):2563. doi: 10.1038/s41467-022-30190-1 35538075 PMC9091225

[10] Banks L, Spence P, Androphy E, Hubbert N, Matlashewski G, Murray A, et al. Identification of human papillomavirus type 18 E6 polypeptide in cells derived from human cervical carcinomas. J Gen Virol. 1987;68 (Pt 5):1351–9. doi: 10.1099/0022-1317-68-5-1351 3033140

[11] Schwarz E, Freese UK, Gissmann L, Mayer W, Roggenbuck B, Stremlau A, et al. Structure and transcription of human papillomavirus sequences in cervical carcinoma cells. Nature. 1985;314(6006):111–4. doi: 10.1038/314111a0 2983228

[12] Zhang C-Y, Bao W, Wang L-H. Downregulation of p16(ink4a) inhibits cell proliferation and induces G1 cell cycle arrest in cervical cancer cells. Int J Mol Med. 2014;33(6):1577–85. doi: 10.3892/ijmm.2014.1731 24714974

[13] Wang J, Sampath A, Raychaudhuri P, Bagchi S. Both Rb and E7 are regulated by the ubiquitin proteasome pathway in HPV-containing cervical tumor cells. Oncogene. 2001;20(34):4740–9. doi: 10.1038/sj.onc.1204655 11498796

[14] Harrington EA, Bruce JL, Harlow E, Dyson N. pRB plays an essential role in cell cycle arrest induced by DNA damage. Proc Natl Acad Sci U S A. 1998;95(20):11945–50. doi: 10.1073/pnas.95.20.11945 9751770 PMC21745

[15] Barbosa MS, Edmonds C, Fisher C, Schiller JT, Lowy DR, Vousden KH. The region of the HPV E7 oncoprotein homologous to adenovirus E1a and Sv40 large T antigen contains separate domains for Rb binding and casein kinase II phosphorylation. EMBO J. 1990;9(1):153–60. doi: 10.1002/j.1460-2075.1990.tb08091.x 2153075 PMC551641

[16] Basukala O, Mittal S, Massimi P, Bestagno M, Banks L. The HPV-18 E7 CKII phospho acceptor site is required for maintaining the transformed phenotype of cervical tumour-derived cells. PLoS Pathog. 2019;15(5):e1007769. doi: 10.1371/journal.ppat.1007769 31116803 PMC6530875

[17] Dizanzo MP, Bugnon Valdano M, Basukala O, Banks L, Gardiol D. Novel effect of the high risk-HPV E7 CKII phospho-acceptor site on polarity protein expression. BMC Cancer. 2022;22(1):1015. doi: 10.1186/s12885-022-10105-5 36153517 PMC9509620

[18] Javorsky A, Humbert PO, Kvansakul M. Viral manipulation of cell polarity signalling. Biochim Biophys Acta Mol Cell Res. 2023;1870(7):119536. doi: 10.1016/j.bbamcr.2023.119536 37437846

[19] Banks L, Pim D, Thomas M. Human tumour viruses and the deregulation of cell polarity in cancer. Nat Rev Cancer. 2012;12(12):877–86. doi: 10.1038/nrc3400 23175122

[20] Dizanzo MP, Marziali F, Brunet Avalos C, Bugnon Valdano M, Leiva S, Cavatorta AL, et al. HPV E6 and E7 oncoproteins cooperatively alter the expression of Disc Large 1 polarity protein in epithelial cells. BMC Cancer. 2020;20(1):293. doi: 10.1186/s12885-020-06778-5 32264889 PMC7137215

[21] Yang Y, Mlodzik M. Wnt-Frizzled/planar cell polarity signaling: cellular orientation by facing the wind (Wnt). Annu Rev Cell Dev Biol. 2015;31:623–46. doi: 10.1146/annurev-cellbio-100814-125315 26566118 PMC4673888

[22] Yang W, Garrett L, Feng D, Elliott G, Liu X, Wang N, et al. Wnt-induced Vangl2 phosphorylation is dose-dependently required for planar cell polarity in mammalian development. Cell Res. 2017;27(12):1466–84. doi: 10.1038/cr.2017.127 29056748 PMC5717403

[23] Dreyer CA, VanderVorst K, Carraway KL. Vangl as a master scaffold for wnt/planar cell polarity signaling in development and disease. Front Cell Dev Biol. 2022;10:887100. doi: 10.3389/fcell.2022.887100 35646914 PMC9130715

[24] VanderVorst K, Dreyer CA, Hatakeyama J, Bell GRR, Learn JA, Berg AL, et al. Vangl-dependent Wnt/planar cell polarity signaling mediates collective breast carcinoma motility and distant metastasis. Breast Cancer Res. 2023;25(1):52. doi: 10.1186/s13058-023-01651-2 37147680 PMC10163820

[25] Zhao L, Wang L, Zhang C, Liu Z, Piao Y, Yan J, et al. E6-induced selective translation of WNT4 and JIP2 promotes the progression of cervical cancer via a noncanonical WNT signaling pathway. Signal Transduct Target Ther. 2019;4:32. doi: 10.1038/s41392-019-0060-y 31637011 PMC6799841

[26] Zhang R, Lu H, Lyu Y-Y, Yang X-M, Zhu L-Y, Yang G-D, et al. E6/E7-P53-POU2F1-CTHRC1 axis promotes cervical cancer metastasis and activates Wnt/PCP pathway. Sci Rep. 2017;7:44744. doi: 10.1038/srep44744 28303973 PMC5356195

[27] Dreyer CA, VanderVorst K, Natwick D, Bell G, Sood P, Hernandez M, et al. A complex of Wnt/planar cell polarity signaling components Vangl1 and Fzd7 drives glioblastoma multiforme malignant properties. Cancer Lett. 2023;567:216280. doi: 10.1016/j.canlet.2023.216280 37336284 PMC10582999

[28] Hatakeyama J, Wald JH, Printsev I, Ho H-YH, Carraway KL. Vangl1 and Vangl2: planar cell polarity components with a developing role in cancer. Endocr Relat Cancer. 2014;21(5):R345-56. doi: 10.1530/ERC-14-0141 24981109 PMC4332879

[29] Zine El Abidine A, Tomaić V, Bel Haj Rhouma R, Massimi P, Guizani I, Boubaker S, et al. A naturally occurring variant of HPV-16 E7 exerts increased transforming activity through acquisition of an additional phospho-acceptor site. Virology. 2017;500:218–25. doi: 10.1016/j.virol.2016.10.023 27829177

[30] Chemes LB, Sánchez IE, Smal C, de Prat-Gay G. Targeting mechanism of the retinoblastoma tumor suppressor by a prototypical viral oncoprotein. Structural modularity, intrinsic disorder and phosphorylation of human papillomavirus E7. FEBS J. 2010;277(4):973–88. doi: 10.1111/j.1742-4658.2009.07540.x 20088881

[31] Feng D, Wang J, Yang W, Li J, Lin X, Zha F, et al. Regulation of Wnt/PCP signaling through p97/VCP-KBTBD7-mediated Vangl ubiquitination and endoplasmic reticulum-associated degradation. Sci Adv. 2021;7(20):eabg2099. doi: 10.1126/sciadv.abg2099 33990333 PMC8121430

[32] Hegde RS, Zavodszky E. Recognition and degradation of mislocalized proteins in health and disease. Cold Spring Harb Perspect Biol. 2019;11(11):a033902. doi: 10.1101/cshperspect.a033902 30833453 PMC6824236

[33] Mallik S, Kundu S. Topology and oligomerization of mono- and oligomeric proteins regulate their half-lives in the cell. Structure. 2018;26(6):869-878.e3. doi: 10.1016/j.str.2018.04.015 29804822

[34] Yasumura M, Hagiwara A, Hida Y, Ohtsuka T. Planar cell polarity protein Vangl2 and its interacting protein Ap2m1 regulate dendritic branching in cortical neurons. Genes Cells. 2021;26(12):987–98. doi: 10.1111/gtc.12899 34626136

[35] Navarro Negredo P, Edgar JR, Wrobel AG, Zaccai NR, Antrobus R, Owen DJ, et al. Contribution of the clathrin adaptor AP-1 subunit µ1 to acidic cluster protein sorting. J Cell Biol. 2017;216(9):2927–43. doi: 10.1083/jcb.201602058 28743825 PMC5584140

[36] Carvajal-Gonzalez JM, Balmer S, Mendoza M, Dussert A, Collu G, Roman A-C, et al. The clathrin adaptor AP-1 complex and Arf1 regulate planar cell polarity in vivo. Nat Commun. 2015;6:6751. doi: 10.1038/ncomms7751 25849195 PMC4515971

[37] Mizuguchi K, Aoki H, Aoyama M, Kawaguchi Y, Waguri-Nagaya Y, Ohte N, et al. Three-dimensional spheroid culture induces apical-basal polarity and the original characteristics of immortalized human renal proximal tubule epithelial cells. Exp Cell Res. 2021;404(1):112630. doi: 10.1016/j.yexcr.2021.112630 33971195

[38] Feigin ME, Akshinthala SD, Araki K, Rosenberg AZ, Muthuswamy LB, Martin B, et al. Mislocalization of the cell polarity protein scribble promotes mammary tumorigenesis and is associated with basal breast cancer. Cancer Res. 2014;74(11):3180–94. doi: 10.1158/0008-5472.CAN-13-3415 24662921 PMC4096808

[39] Peglion F, Etienne-Manneville S. Cell polarity changes in cancer initiation and progression. J Cell Biol. 2024;223(1):e202308069. doi: 10.1083/jcb.202308069 38091012 PMC10720656

[40] Laumonnerie C, Solecki DJ. Regulation of polarity protein levels in the developing central nervous system. J Mol Biol. 2018;430(19):3472–80. doi: 10.1016/j.jmb.2018.05.036 29864442 PMC6783131

[41] Hall AHS, Alexander KA. RNA interference of human papillomavirus type 18 E6 and E7 induces senescence in HeLa cells. J Virol. 2003;77(10):6066–9. doi: 10.1128/jvi.77.10.6066-6069.2003 12719599 PMC154052

[42] Barbosa MS, Schlegel R. The E6 and E7 genes of HPV-18 are sufficient for inducing two-stage in vitro transformation of human keratinocytes. Oncogene. 1989;4(12):1529–32. 2556677

[43] Mittal S, Banks L. Molecular mechanisms underlying human papillomavirus E6 and E7 oncoprotein-induced cell transformation. Mutat Res Rev Mutat Res. 2017;772:23–35. doi: 10.1016/j.mrrev.2016.08.001 28528687

[44] Butz K, Ristriani T, Hengstermann A, Denk C, Scheffner M, Hoppe-Seyler F. siRNA targeting of the viral E6 oncogene efficiently kills human papillomavirus-positive cancer cells. Oncogene. 2003;22(38):5938–45. doi: 10.1038/sj.onc.1206894 12955072

[45] Paramore SV, Trenado-Yuste C, Sharan R, Nelson CM, Devenport D. Vangl-dependent mesenchymal thinning shapes the distal lung during murine sacculation. Dev Cell. 2024;59(10):1302-1316.e5. doi: 10.1016/j.devcel.2024.03.010 38569553 PMC11111357

[46] Anastas JN, Biechele TL, Robitaille M, Muster J, Allison KH, Angers S, et al. A protein complex of SCRIB, NOS1AP and VANGL1 regulates cell polarity and migration, and is associated with breast cancer progression. Oncogene. 2012;31(32):3696–708. doi: 10.1038/onc.2011.528 22179838 PMC3419983

[47] Wald JH, Hatakeyama J, Printsev I, Cuevas A, Fry WHD, Saldana MJ, et al. Suppression of planar cell polarity signaling and migration in glioblastoma by Nrdp1-mediated Dvl polyubiquitination. Oncogene. 2017;36(36):5158–67. doi: 10.1038/onc.2017.126 28481871 PMC5589482

[48] van der Net A, Rahman Z, Bordoloi AD, Muntz I, Ten Dijke P, Boukany PE, et al. EMT-related cell-matrix interactions are linked to states of cell unjamming in cancer spheroid invasion. iScience. 2024;27(12):111424. doi: 10.1016/j.isci.2024.111424 39717087 PMC11665421

[49] Jessen TN, Jessen JR. VANGL2 protein stability is regulated by integrin αv and the extracellular matrix. Exp Cell Res. 2019;374(1):128–39. doi: 10.1016/j.yexcr.2018.11.017 30472097 PMC6295236

[50] Kuczek DE, Larsen AMH, Thorseth M-L, Carretta M, Kalvisa A, Siersbæk MS, et al. Collagen density regulates the activity of tumor-infiltrating T cells. J Immunother Cancer. 2019;7(1):68. doi: 10.1186/s40425-019-0556-6 30867051 PMC6417085

[51] Boot RC, Koenderink GH, Boukany PE. Spheroid mechanics and implications for cell invasion. Advances in Physics: X. 2021;6(1). doi: 10.1080/23746149.2021.1978316

[52] Khalil AA, Friedl P. Determinants of leader cells in collective cell migration. Integr Biol (Camb). 2010;2(11–12):568–74. doi: 10.1039/c0ib00052c 20886167

[53] Nguyen-Ngoc K-V, Cheung KJ, Brenot A, Shamir ER, Gray RS, Hines WC, et al. ECM microenvironment regulates collective migration and local dissemination in normal and malignant mammary epithelium. Proc Natl Acad Sci U S A. 2012;109(39):E2595-604. doi: 10.1073/pnas.1212834109 22923691 PMC3465416

[54] Helmlinger G, Netti PA, Lichtenbeld HC, Melder RJ, Jain RK. Solid stress inhibits the growth of multicellular tumor spheroids. Nat Biotechnol. 1997;15(8):778–83. doi: 10.1038/nbt0897-778 9255794

[55] Mark C, Grundy TJ, Strissel PL, Böhringer D, Grummel N, Gerum R, et al. Collective forces of tumor spheroids in three-dimensional biopolymer networks. Elife. 2020;9:e51912. doi: 10.7554/eLife.51912 32352379 PMC7192581

[56] Davey CF, Moens CB. Planar cell polarity in moving cells: think globally, act locally. Development. 2017;144(2):187–200. doi: 10.1242/dev.122804 28096212 PMC5394761

[57] Muñoz-Soriano V, Belacortu Y, Paricio N. Planar cell polarity signaling in collective cell movements during morphogenesis and disease. Curr Genomics. 2012;13(8):609–22. doi: 10.2174/138920212803759721 23730201 PMC3492801

[58] Huang Y, Winklbauer R. Cell cortex regulation by the planar cell polarity protein Prickle1. J Cell Biol. 2022;221(7):e202008116. doi: 10.1083/jcb.202008116 35512799 PMC9082893

[59] Bastock R, Strutt D. The planar polarity pathway promotes coordinated cell migration during Drosophila oogenesis. Development. 2007;134(17):3055–64. doi: 10.1242/dev.010447 17652348 PMC1991286

[60] Gandalovičová A, Vomastek T, Rosel D, Brábek J. Cell polarity signaling in the plasticity of cancer cell invasiveness. Oncotarget. 2016;7(18):25022–49. doi: 10.18632/oncotarget.7214 26872368 PMC5041887

[61] Desmaison A, Frongia C, Grenier K, Ducommun B, Lobjois V. Mechanical stress impairs mitosis progression in multi-cellular tumor spheroids. PLoS One. 2013;8(12):e80447. doi: 10.1371/journal.pone.0080447 24312473 PMC3848935

[62] Dolega ME, Delarue M, Ingremeau F, Prost J, Delon A, Cappello G. Cell-like pressure sensors reveal increase of mechanical stress towards the core of multicellular spheroids under compression. Nat Commun. 2017;8:14056. doi: 10.1038/ncomms14056 28128198 PMC5290143

[63] Delarue M, Montel F, Vignjevic D, Prost J, Joanny J-F, Cappello G. Compressive stress inhibits proliferation in tumor spheroids through a volume limitation. Biophys J. 2014;107(8):1821–8. doi: 10.1016/j.bpj.2014.08.031 25418163 PMC4213738

[64] Cheng G, Tse J, Jain RK, Munn LL. Micro-environmental mechanical stress controls tumor spheroid size and morphology by suppressing proliferation and inducing apoptosis in cancer cells. PLoS One. 2009;4(2):e4632. doi: 10.1371/journal.pone.0004632 19247489 PMC2645686

[65] Adachi Y, Kimura R, Hirade K, Yanase S, Nishioka Y, Kasuga N, et al. Scribble mis-localization induces adaptive resistance to KRAS G12C inhibitors through feedback activation of MAPK signaling mediated by YAP-induced MRAS. Nat Cancer. 2023;4(6):829–43. doi: 10.1038/s43018-023-00575-2 37277529

[66] Wang N, Song L, Xu Y, Zhang L, Wu Y, Guo J, et al. Loss of Scribble confers cisplatin resistance during NSCLC chemotherapy via Nox2/ROS and Nrf2/PD-L1 signaling. EBioMedicine. 2019;47:65–77. doi: 10.1016/j.ebiom.2019.08.057 31495720 PMC6796531

[67] Shi H, Selvamani SP, Zelei R, Zhuang L, Wang D, Johnson B, et al. Validation of chemoresistance phenotypes in pleural mesothelioma across 2D, 3D, and in vivo models. Sci Rep. 2026;16(1):8396. doi: 10.1038/s41598-026-38692-4 41673140 PMC12972125

[68] Senrung A, Lalwani S, Janjua D, Tripathi T, Kaur J, Ghuratia N, et al. 3D tumor spheroids: morphological alterations a yardstick to anti-cancer drug response. In Vitro Model. 2023;2(6):219–48. doi: 10.1007/s44164-023-00059-8 39872501 PMC11756486

[69] Ma T, Li B, Wang R, Lau PK, Huang Y, Jiang L, et al. A mechanism for differential sorting of the planar cell polarity proteins Frizzled6 and Vangl2 at the trans-Golgi network. J Biol Chem. 2018;293(22):8410–27. doi: 10.1074/jbc.RA118.001906 29666182 PMC5986227

[70] Humphries AC, Molina-Pelayo C, Sil P, Hazelett CC, Devenport D, Mlodzik M. A Van Gogh/Vangl tyrosine phosphorylation switch regulates its interaction with core Planar Cell Polarity factors Prickle and Dishevelled. PLoS Genet. 2023;19(7):e1010849. doi: 10.1371/journal.pgen.1010849 37463168 PMC10381084

[71] Torres MA, Nelson WJ. Colocalization and redistribution of dishevelled and actin during Wnt-induced mesenchymal morphogenesis. J Cell Biol. 2000;149(7):1433–42. doi: 10.1083/jcb.149.7.1433 10871283 PMC2175133

[72] Hu W, Li M, Wu J, Chen H, Zhao T, Zhang C, et al. Inhibition of Dishevelled-2 suppresses the biological behavior of pancreatic cancer by downregulating Wnt/β-catenin signaling. Oncol Lett. 2021;22(5):769. doi: 10.3892/ol.2021.13030 34589148 PMC8442142

[73] Hao C-C, Xu C-Y, Zhao X-Y, Luo J-N, Wang G, Zhao L-H, et al. Up-regulation of VANGL1 by IGF2BPs and miR-29b-3p attenuates the detrimental effect of irradiation on lung adenocarcinoma. J Exp Clin Cancer Res. 2020;39(1):256. doi: 10.1186/s13046-020-01772-y 33228740 PMC7687693

[74] Feng X in. Core planar cell polarity genes VANGL1 and VANGL2 in predisposition to congenital vertebral malformations. Proceedings of the National Academy of Sciences of the United States of America. 2024. 10.1073/pnas.2310283121 PMC11067467 38669183

[75] Lin C-H, Chang H-S, Yu WCY. USP11 stabilizes HPV-16E7 and further modulates the E7 biological activity. J Biol Chem. 2008;283(23):15681–8. doi: 10.1074/jbc.M708278200 18408009 PMC3259633

[76] Westrich JA, Warren CJ, Klausner MJ, Guo K, Liu C-W, Santiago ML, et al. Human Papillomavirus 16 E7 Stabilizes APOBEC3A Protein by Inhibiting Cullin 2-Dependent Protein Degradation. J Virol. 2018;92(7):e01318-17. doi: 10.1128/JVI.01318-17 29367246 PMC5972886

[77] Xia C, Xiao C, Luk HY, Chan PKS, Boon SS. The ubiquitin specific protease 7 stabilizes HPV16E7 to promote HPV-mediated carcinogenesis. Cell Mol Life Sci. 2023;80(10):278. doi: 10.1007/s00018-023-04941-2 37682346 PMC11072444

[78] Gbala I, Kavcic N, Banks L. The retinoblastoma protein contributes to maintaining the stability of HPV E7 in cervical cancer cells. J Virol. 2025;99(4). doi: 10.1128/jvi.02203-24 PMC11998531 40130877

[79] Wu J-S, Jiang J, Chen B-J, Wang K, Tang Y-L, Liang X-H. Plasticity of cancer cell invasion: Patterns and mechanisms. Transl Oncol. 2021;14(1):100899. doi: 10.1016/j.tranon.2020.100899 33080522 PMC7573380

[80] Pim D, Massimi P, Dilworth SM, Banks L. Activation of the protein kinase B pathway by the HPV-16 E7 oncoprotein occurs through a mechanism involving interaction with PP2A. Oncogene. 2005;24(53):7830–8. doi: 10.1038/sj.onc.1208935 16044149

[81] Gonzalez SL, Stremlau M, He X, Basile JR, Münger K. Degradation of the retinoblastoma tumor suppressor by the human papillomavirus type 16 E7 oncoprotein is important for functional inactivation and is separable from proteasomal degradation of E7. J Virol. 2001;75(16):7583–91. doi: 10.1128/JVI.75.16.7583-7591.2001 11462030 PMC114993

[82] Young LC, Hartig N, Muñoz-Alegre M, Oses-Prieto JA, Durdu S, Bender S, et al. An MRAS, SHOC2, and SCRIB complex coordinates ERK pathway activation with polarity and tumorigenic growth. Mol Cell. 2013;52(5):679–92. doi: 10.1016/j.molcel.2013.10.004 24211266

[83] Chen C, Okayama H. High-efficiency transformation of mammalian cells by plasmid DNA. Mol Cell Biol. 1987;7(8):2745–52. doi: 10.1128/mcb.7.8.2745-2752.1987 3670292 PMC367891

[84] Tomaić V, Gardiol D, Massimi P, Ozbun M, Myers M, Banks L. Human and primate tumour viruses use PDZ binding as an evolutionarily conserved mechanism of targeting cell polarity regulators. Oncogene. 2009;28(1):1–8. doi: 10.1038/onc.2008.365 18820705 PMC3569523

